# Large-scale visualization of α-synuclein oligomers in Parkinson’s disease brain tissue

**DOI:** 10.1038/s41551-025-01496-4

**Published:** 2025-10-01

**Authors:** Rebecca Andrews, Bin Fu, Christina E. Toomey, Jonathan C. Breiter, Joanne Lachica, Joseph S. Beckwith, Ru Tian, Emma E. Brock, Lisa-Maria Needham, Gregory J. Chant, Camille Loiseau, Angèle Deconfin, Kenza Baspin, Rebeka Popovic, James Evans, Yen Goh, Begüm Kurt, Lenart Senicar, Marisa Edmonds, Tim Bartels, Nora Bengoa-Vergniory, Peter J. Magill, Zane Jaunmuktane, Oliver J. Freeman, Benjamin J. M. Taylor, John Hardy, Tammaryn Lashley, Mina Ryten, Michele Vendruscolo, Nicholas W. Wood, Lucien E. Weiss, Sonia Gandhi, Steven F. Lee

**Affiliations:** 1 Yusuf Hamied Department of Chemistry, University of Cambridge, Cambridge, UK; 2 Aligning Science Across Parkinson’s Collaborative Research Network, Chevy Chase, MD USA; 3 Oncology R&D, AstraZeneca, Cambridge, UK; 4 Department of Clinical and Movement Neurosciences, UCL Queen Square Institute of Neurology, London, UK; 5 The Francis Crick Institute, London, UK; 6 Centre for Misfolding Diseases, Yusuf Hamied Department of Chemistry, University of Cambridge, Cambridge, UK; 7 Medical Research Council Brain Network Dynamics Unit, Nuffield Department of Clinical Neurosciences, University of Oxford, Oxford, UK; 8 Department of Engineering Physics, Polytechnique Montréal, Montréal, Québec Canada; 9 Achucarro Basque Center for Neuroscience, Leioa, Spain; 10 UK Dementia Research Institute, University College London, London, UK; 11 Ikerbasque-Basque Foundation for Science, Bilbao, Spain; 12 Department of Neuroscience, University of the Basque Country, Leioa, Spain; 13 Division of Neuropathology, National Hospital for Neurology and Neurosurgery, University College London NHS Foundation Trust, London, UK; 14 Neuroscience BioPharmaceuticals R&D, AstraZeneca, Cambridge, UK; 15 Department of Neurodegenerative Diseases, UCL Queen Square Institute of Neurology, London, UK; 16 UK Dementia Research Institute, University of Cambridge, Cambridge, UK; 17 Great Ormond Street Institute of Child Health, University College London, London, UK; 18 Department of Clinical Neurosciences, School of Clinical Medicine, University of Cambridge, Cambridge, UK; 19 Present Address: Department of Chemistry, University of Zurich, Zurich, Switzerland; 20 Present Address: Cambridge Advanced Imaging Centre, University of Cambridge, Cambridge, UK; 21 Present Address: MSD R&D Innovation Centre, London, UK

**Keywords:** High-throughput screening, Nanoscale biophysics

## Abstract

Parkinson’s disease (PD) is a neurodegenerative condition characterized by the presence of intraneuronal aggregates containing fibrillar ɑ-synuclein known as Lewy bodies. These large end-stage species are formed by smaller soluble protein nanoscale assemblies, often termed oligomers, which are proposed as early drivers of pathogenesis. Until now, this hypothesis has remained controversial, at least in part because it has not been possible to directly visualize nanoscale assemblies in human brain tissue. Here we present Advanced Sensing of Aggregates—Parkinson’s Disease, an imaging method to generate large-scale α-synuclein aggregate maps in post-mortem human brain tissue. We combined autofluorescence suppression with single-molecule fluorescence microscopy, which together enable the detection of nanoscale α-synuclein aggregates. To demonstrate the use of this platform, we analysed ~1.2 million nanoscale aggregates from the anterior cingulate cortex in human post-mortem brain samples from patients with PD and healthy controls. Our data reveal a disease-specific shift in a subpopulation of nanoscale assemblies that represent an early feature of the proteinopathy that underlies PD. We anticipate that quantitative information about this distribution provided by Advanced Sensing of Aggregates—Parkinson’s Disease will enable mechanistic studies to reveal the pathological processes caused by α-synuclein aggregation.

## Abstract (web summary)

An optical detection and analysis platform quantifies aggregate density, distribution and size in fluorescently labelled post-mortem human brain tissue.

## Main

Parkinson’s disease (PD) is a progressive neurodegenerative disorder that initially causes the loss of dopaminergic neurons in the substantia nigra, resulting in a movement disorder consisting of tremors, bradykinesia and rigidity^1^. The disease spreads over several years, affecting multiple brain regions and resulting in dementia, neuropsychiatric, autonomic and sleep disturbances^2^. Pathologically, PD is characterized by neuronal loss accompanied by the accumulation of microscale α-synuclein aggregates called Lewy bodies and Lewy neurites. The morphologies of these structures are typically either neuritic (~5–10 µm in length) or round (~5–20 µm diameter)^3^, and have been observed in varying densities in different brain regions depending on the disease stage^4^. Such structures form the basis of PD diagnostic staging criteria^4,5^. Further evidence implicating α-synuclein in PD arises from the observation that mutations or gene rearrangements in *SNCA*^6–13^, the gene encoding the α-synuclein protein, cause early-onset autosomal dominant PD and variants in the *SNCA* gene increase the risk of sporadic PD.

Protein aggregation occurs through the self-assembly of monomeric α-synuclein into small protein assemblies, which undergo growth and structural conversion to soluble intermediate species, gradually acquiring cross β-sheet structure^14–16^. The smaller intermediary structures, including small fibrillar species and amorphous oligomers^17^, bridge monomers and the much larger fibrillar structures found in Lewy bodies^18^, are expected to contain tens to hundreds of monomeric protein units^19^. Oligomers can have a variety of post-translational modifications and conformations and contain α-helices and/or β-pleated sheets^20–22^. In cell culture and animal models, it has been shown that oligomers cause neurotoxicity and neuronal death consistent with PD^23–30^. Oligomers have mainly been studied using recombinant protein, but these aggregates are not identical to the oligomeric assemblies found in human tissue, and different preparation protocols for recombinant oligomers can lead to a variety of characteristics^18,31–33^, motivating studies on native proteins; proteins that are in their natural structure and functional confirmation. Detecting endogenous, small aggregates in a post-mortem human brain has long remained elusive, primarily owing to a lack of sensitivity. Proximity-ligation assays (PLA) have verified the presence of small α-synuclein aggregates by signal amplification^16,34^. However, direct visualization of nanoscale assemblies in brain tissue has, so far, not been possible, hindering our understanding of how these species are distributed spatially and by size.

Here, we present an optical detection and analysis platform, Advanced Sensing of Aggregates–Parkinson’s Disease (ASA–PD), that can be used to quantify aggregate density, distribution and size directly in fluorescently labelled post-mortem human brain tissue. We applied ASA–PD to characterize α-synuclein assemblies in large areas of post-mortem tissue sections from patients with PD and matched healthy controls (HCs). By enhancing the sensitivity of traditional immunofluorescence techniques, combined with our analytical approach, we have been able to detect and characterize over 1.2 million α-synuclein aggregates. The entire dataset, metadata and analysis toolset have been made available online (Data Availability^35,36^). In addition to the microscale aggregates described by classical Lewy pathology^37^, our data show that assemblies are present in both PD and HC samples. Notably, PD samples contained a shifted subpopulation of bright nanoscale assemblies largely absent from the HCs. The presence of this ‘disease-specific’ shift was detected in PD cases from different brain banks, disease stages, immunofluorescent labels and antigen-retrieval methods. The presence of this subpopulation was confirmed using a range of orthogonal analytical approaches. Our findings are consistent with the hypothesis that misfolded α-synuclein readily form a continuum of larger nanoscale aggregates that eventually give rise to the microscale structures traditionally associated with the disease. We visualized and quantified the distributions of measured species in brain tissue, characterized their biochemical properties and determined their distributions in relation to several specific cell types in the human brain.

## Results

### Autofluorescence suppression and high-sensitivity microscopy reveals nanoscale assemblies in human brain tissue

**Fig. 1:**
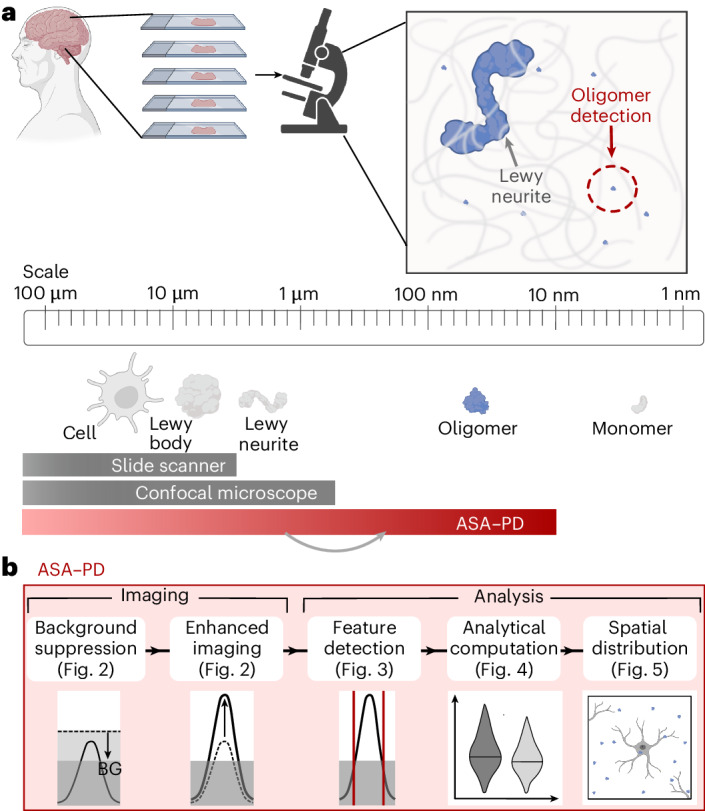
ASA–PD. **a**, ASA-PD is an imaging and analysis method for detecting protein aggregates in tissue down to nanoscale aggregates. **b**, The five main steps for imaging and analysis. Background suppression and enhanced imaging improve the signal-to-noise ratio such that aggregates can be detected and quantified in the analysis pipeline, including the spatial distributions relative to cell-specific markers. BG, background. Panels **a** and **b** created with BioRender.com.

An overview of the ASA–PD pipeline is shown in Fig. 1. In brief, the aim is to capture spatial data over the entire scale of structure sizes most critical in PD, from individual cells to small aggregates (Fig. 1a). Detailed descriptions of the sample preparation steps are described in the Methods. First, 8-μm-thick brain tissue sections were mounted on glass slides, stained and then processed in the five stages illustrated in Fig. 1b: (1) background suppression, (2) enhanced imaging, (3) feature detection, (4) analytical computation and (5) spatial distribution analysis, where the first two steps contain the experimental portion of our workflow and steps 3–5 perform image-processing tasks and analysis.

**Fig. 2:**
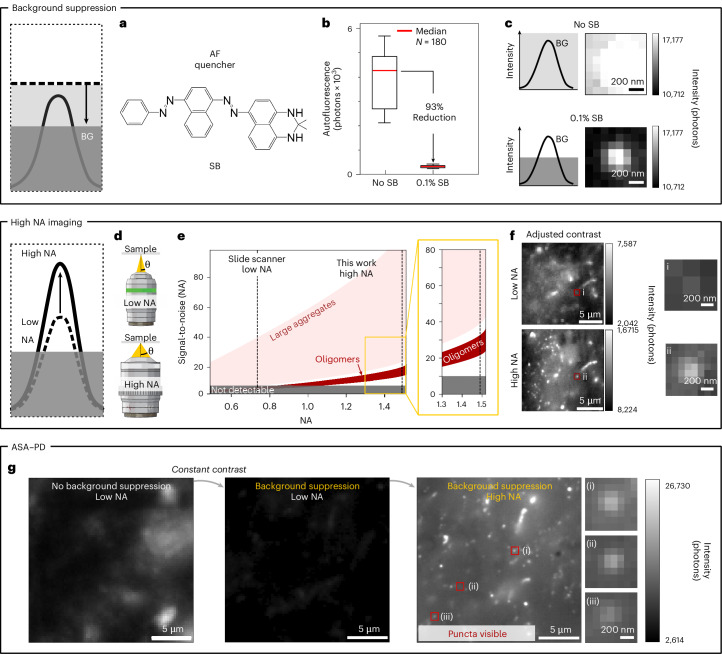
Background suppression and high-sensitivity microscopy in human brain tissue. **a**, Background suppression is achieved with the autofluorescence quencher Sudan Black (SB). **b**, Box plots showing the IQR and 5th–95th percentile bounds of autofluorescence (AF) intensity before (median of 4,400) and after treatment with 0.1% SB (median of 333). *N* = 180 images per sample. **c**, Before quenching, the fluorescence from Alexa Fluor 568 labelled small aggregates is masked by the background autofluorescence. After quenching, small aggregates can be easily visualized (both images excited at 561 nm, 26 W cm^−2^). **d**, A high NA objective collects a larger amount of light from the sample. **e**, The modelled signal-to-noise ratio for imaging punctate objects in post-quenched tissue background at 100*×* magnification across a range of NAs of objectives. Only at high NA (>1) large aggregates and oligomers become detectable. **f**, Images of p-syn stained PD tissue with 40× magnification, NA = 0.75 (top) and 100*×*, NA = 1.49 (bottom). Close-ups show that the same small aggregate is clearly visible at high magnification and high NA. **g**, Images of p-syn stained PD tissue with no background suppression and low NA (left), background suppression implemented and low NA (middle) and background suppression implemented and high NA (right). Several example puncta are shown in the closeups (oligomers) after background suppression is implemented and a high NA objective is used.

Observing protein aggregates is relatively routine in in vitro conditions^38–40^, but detecting small species in vivo poses a challenge owing to the poor signal-to-noise ratio in tissue. High background intensity, caused by tissue autofluorescence, acts as a noise floor that obscures the presence of dim objects such as oligomeric species. This noise effectively implements a brightness filter that leaves only large protein aggregates, with many attached fluorescent antibodies, as detectable species. To reduce the high autofluorescence of human brain tissue that inhibits sensitive imaging, we deployed Sudan Black B (Fig. 2a), a fat-soluble diazo dye and well-known autofluorescence quencher on untreated brain tissue sections^41^. Under optimized conditions, 10 min of incubation with 0.1% Sudan Black led to a 93% reduction in background autofluorescence for 561 nm laser excitation (26 W cm^−2^ illumination intensity), corresponding to a decrease in median detected photon counts from 4,400 ± 1,040 photons ± median absolute deviation (MAD) to 333 ± 47 photons (Fig. 2b) (the background reduction for other excitation colours (488, 561 nm), treatment times and concentrations are shown in Supplementary Fig. 1). Next, we repeated this background suppression step on antibody-labelled samples and evaluated various antibodies against multiple forms of α-synuclein for specificity and detectability (Supplementary Figs. 2 and 3 and Supplementary Table 1). The background reduction by Sudan Black facilitated the reliable detection of small features in images with a vastly improved signal-to-noise ratio for some of the antibodies tested, as shown in Fig. 2c.

To visualize the α-synuclein aggregates most associated with PD, we used an antibody targeting phosphorylated α-synuclein at serine 129 (hereafter called p-syn). This post-translational modification promotes inclusion formation and/or toxicity in human cells^42^, *Drosophila*^43^ and rodent models^44,45^. Further, p-syn forms the vast majority of all insoluble α-synuclein aggregates in the PD brain^46^. Given this link between p-syn and pathology in synucleinopathies, we tested a variety of antibodies (Supplementary Table 1), including two complementary antibodies targeting the pS129 epitope of α-synuclein raised in two species (rabbit, AB_2270761, and mouse, AB_2819037). The AB_2819037 antibody showed characteristic Lewy pathology in both DAB and immunofluorescence staining and were shown to be specific through substantial co-localization with a second antibody for total α-synuclein (AB_2832854) (Supplementary Fig. 2). The final p-syn antibody selection, AB_2819037, was selected because of (1) the degree of coincidence of the p-syn antibody compared with total α-synuclein; (2) co-localization with other disease-related proteins, such as ubiquitin and p62; and (3) the demonstration of antibody specificity for human α-synuclein based on mouse tissue with the overexpression or knockout of human α-synuclein^47^. Furthermore, we confirmed (via electron microscopy and fluorescence imaging) that purified p-syn can aggregate in vitro, and form β-sheet rich 10-nm assemblies^48^. These recombinant p-syn protein aggregates can be detected by the same AB_2819037 antibody (Supplementary Fig. 4).

One way to improve the signal-to-noise ratio beyond reducing the overall background intensity is by improving the light-collection efficiency of the imaging system. In most microscopes, the least efficient step in light collection occurs at the objective lens of the microscope and is encoded in the numerical aperture (NA). Using a high NA objective lens has two main impacts: first, the NA scales with the collection angle of collected light^49^ (Fig. 2d and Supplementary equation (3)), and thus, more photons from the sample are collected at high NA. Second, increasing the NA improves the image resolution^50^ (Supplementary equation (8)). For imaging in tissue, we deployed a 1.49 NA oil-immersion, 100× microscope objective lens often used in single-molecule fluorescence applications^51^. The result is an overall increase in the signal-to-noise ratio for all objects, which is particularly important for the nanoscale assemblies that fall below the detectability range for lower NA objectives (Fig. 2e), such as the air objectives most often used in slide scanners for clinical applications^52^. Figure 2f compares a 0.75 NA 40× air objective lens (top) with the 1.49 NA 100× oil objective lens used in this study (bottom) for the same tissue sample stained for phosphorylated α-synuclein and quenched with 0.1% Sudan Black. The effect of background suppression and increased light collection using a high NA are shown in Fig. 2g. In these images, a wide variety of object sizes become visible; which we divide into two classes on the basis of their apparent size relative to the diffraction limit. Specifically, we define ‘large’ as greater than the optical diffraction limit of visible light, spanning ~200 nm to tens of microns, and ‘nanoscale’ as objects below the optical diffraction limit (<200 nm). The fluorescence signal from the latter manifests as small symmetric puncta in the image. Three examples of the nanoscale objects that become visible via ASA–PD are highlighted in Fig. 2g(i–iii). We refer to the latter objects as protein assemblies.

Applying the ASA–PD protocol within tissue revealed hundreds of detectable fluorescent puncta per field of view (FOV) (55 × 55 µm^2^) in both PD and HC samples (Supplementary Fig. 5). To perform statistically robust comparisons between samples, we developed a computationally efficient method for detecting and quantifying fluorescent species. This open-source analysis pipeline^53^ facilitates the rapid processing of large image libraries, facilitating transparent, shareable and verifiable results.

**Fig. 3:**
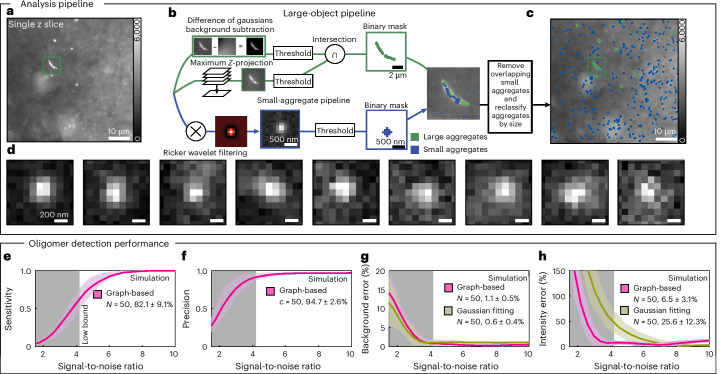
Aggregate detection pipeline. **a**, A typical sample image containing features of various sizes and intensities, that is, Lewy neurites, micron-scale aggregates and sub-diffraction-limit oligomers. **b**, The aggregate detection pipeline for measuring large aggregates (top) and subdiffraction-sized features (bottom). The large object pipeline combines the z-projected intensity data with background-subtracted and threshold individual slices to generate a binary mask. Small aggregates are identified using a Ricker wavelet filter that acts as a bandpass, emphasizing small spots, which are then measured with a threshold and sorted by the number of pixels above the background. Features larger than the diffraction limit are reclassified as ‘large’ and features overlapping between the two masks are removed from the small aggregate pool. **c**, The large (green) and oligomer (blue) masks shown over the original image. **d**, The representative oligomers detected from **c**. **e**–**h**, The quantification of the pipeline performance using simulated images of diffraction-limited spots on a noisy background at various signal-to-noise ratios. The grey shaded region represents the lower quartile determined from experimental conditions, while the green and pink shaded area represents the mean ± s.d. The intensity and average background values for all detected peaks in simulated images were estimated by quantifying the pixel values around the detected peaks (pink curve) and by fitting a symmetric two-dimensional-Gaussian function with nonlinear least squares fitting (green). The presented values were obtained by averaging the mean and s.d. within the IQR of the experimental CNR data (Q1 = 4.2, Q3 = 8.1).

A schematic illustrating the analysis method and its validation is shown in Fig. 3. In brief, the analysis pipeline identifies features in an image, classifies them as either large aggregates or protein assemblies and quantifies details such as brightness, size and position^53^. A detailed description of the analysis is provided in Supplementary Information Note 3.1 and Supplementary Figs. 6–12. Figure 3a shows a typical PD image containing nano and microscale features. Microscale aggregates, such as Lewy bodies and Lewy neurites, are extremely bright in the dataset. These objects can be segmented with a simple intensity threshold after a background subtraction step (large-object pipeline in Fig. 3b and Supplementary Fig. 7). As large objects sometimes extend over multiple z-slices, the mask in each plane is multiplied by a segmented maximum-intensity projection from the z-stack. Smaller aggregates appear as dim, diffraction-limited puncta, and it is crucial to account for local background heterogeneity for detection (small-aggregate pipeline in Fig. 3b). To do so, we applied a bandpass filter to each image which selects features on the scale of the diffraction limit^54^ (Supplementary Figs. 6 and 9). Next, a threshold was used to create a mask containing only small objects. Objects with a footprint larger than the diffraction limit^49^ were reclassified as ‘large’ for subsequent analysis. Finally, the large and small aggregate masks were compared, and overlapping objects were removed from the nanoscale object dataset. Figure 3c shows an overlay of the detected objects on the original image, and a gallery of diffraction-limited puncta is shown in Fig. 3d.

To evaluate the performance of the pipeline for detecting and characterizing nanoscale assemblies, we simulated images of puncta on noisy backgrounds at various signal-to-noise levels based on empirically determined parameters (Supplementary Note 3.2, Simulations). In the signal-to-noise range of our data, approximately ~4–8, the algorithm’s sensitivity was >82%, with a precision of >94% (Fig. 3e,f). At the same time, the relative error for estimating the local background per puncta outperformed nonlinear least-squares Gaussian fitting in this low signal-to-noise regime where Gaussian fitting performed poorly on aberrantly detected pixels, that is, false positives (Fig. 3g,h).

### ASA–PD reveals a disease-specific shift in the nanoscale population of α-synuclein assemblies

**Fig. 4:**
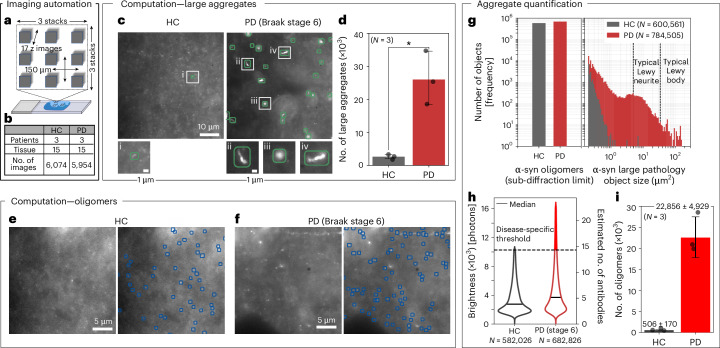
Aggregate distributions in human brain tissue. **a**, The imaging of grey matter was performed in three areas, each area being a 3 × 3 grid of z stacks (17 slices) spaced 150 µm apart. **b**, The number of HCs and patients with PD (*n* = 3), number of tissue sections (*n* = 15) and number of images taken *N*_HC_ = 6,074 and *N*_PD_ = 5,954. **c**, Examples of analysed FOVs showing only the detected large aggregates. **d**, The number of large aggregates detected per patient over 1,800 FOVs (5.4 mm^2^). The mean ± s.d. for large aggregates was 3,866 ± 408 in HCs and 26,314 ± 7712 in PD, the means were compared by a two-tailed two-sample *t*-test, with *P* = 0.0147. **e**,**f**, Example FOVs of detected α-synuclein oligomers in HC and PD (Braak stage 6), respectively. **g**, The total number of α-synuclein aggregates in HC and PD tissues. The left panel shows oligomers (<0.04 μm^2^). The right panel shows large aggregates (>0.04 μm^2^). The typical Lewy neurites sizes (~5–30 μm^2^) and Lewy bodies (~30–300 μm^2^) are shown for reference. **h**, Violin plot of brightness of detected oligomers truncated at 1.5× IQR. Oligomers in HC had a median of 2,750 photons (MAD of 1,060) and of 3,700 photons (MAD of 1,690) for PD. The bright subpopulation of oligomers is shown in red for PD. **i**, The total number of detected oligomers per patient above this brightness threshold, 10,280 photons. Error bars are variation in boundary rejection percentage per patient, propagated. *P < 0.05.

To characterize the distributions of α-synuclein in brain tissue, we selected three PD brains (Braak stage 6) and three HC brains for imaging (Supplementary Table 2). Tissue sections from the anterior cingulate cortical gyrus were put through the ASA–PD process. At this point, three principal regions within the grey matter were selected for investigation. At each of these regions, nine FOVs were captured in a 3 × 3 grid with a lateral separation of 150 μm to avoid any spatial overlap (each image covers 55 × 55 µm^2^). In total, 17 axial planes were recorded using a 500 nm step size (Fig. 4a and Methods). This process generated 13,770 high-resolution images (>41.6 mm^2^) that were manually validated to ensure the sample was in focus and the tissue contained no notable tears or defects. After this verification step, 12,028 images remained, 87.5% of the original dataset (5,954 PD and 6,074 HC images). These images were analysed as described in the previous section to map large aggregates (Fig. 4c,d) and nanoscale assemblies (Fig. 4e,f). Negative control samples, lacking primary antibodies, were also tested using PD tissue to quantify the degree of false positives caused by residual autofluorescence and unbound secondary antibodies (Supplementary Fig. 12).

From the ~12,000 images recorded across 30 tissue sections, we obtained a dataset containing more than 125,000 large aggregates and 1,260,000 nanoscale assemblies^35,36^. From the ~400 FOVs (~1.2 mm^2^), from each patient sample, the average number of large aggregates detected was ~tenfold higher in patients with PD than in the HC, with 26,314 ± 7712 in PD and 3,866 ± 408 in HC, respectively (Fig. 4d). These aggregates were distributed over a broad range of sizes from 0.04 to 100 µm^2^ in PD and 0.04 to 1 µm^2^ in HC (Fig. 4g), where the aggregate sizes associated with Lewy pathology essentially exclusively found in PD samples, consistent with the original tissue classifications (Supplementary Table 2). The total number of detected nanoscale objects in PD and HCs were much more similar (Fig. 4g), with 682,826 and 582,026 objects, respectively (with densities 0.082 objects per μm^2^ for PD and 0.067 objects per μm^2^ for HC).

While image resolution remains fundamentally diffraction-limited, the high sensitivity of ASA–PD to dim puncta, coupled with their relative sparsity, allows the detection of ~10 nm objects—far below the diffraction limit. At this scale, the resolution obscures aggregate sizes; however, for larger aggregates, where the size and brightness can be measured, the two were strongly linearly proportional, *R*^2^ > 0.99 (Supplementary Fig. 13a). We, therefore, characterized the distribution of nanoscale-object intensities as a proxy for the approximate size of these assemblies (Fig. 4h). The brightness distributions for all measured objects are shown in Supplementary Fig. 13b together with the estimated number of bound secondary antibodies, assuming each antibody contributes ~700 photons in our imaging conditions (Supplementary Fig. 14). Relative to the HC, the median was larger for PD samples, 3,700 photons (MAD of 1,690) and 2,750 photons (MAD of 1,060), and the distribution of brightnesses in PD samples was also broader, characterized by its interquartile range (IQR) IQR_PD_ = 4,280 photons compared with IQR_HC_ = 2,690 for HCs. To determine if the distribution tail was reproducibly different between PD and HC samples, we defined a brightness threshold using the HC measurements (Fig. 4h). The number of nanoscale objects above this threshold (10,280 photons, equating to ~15 bound secondary antibodies) is shown in Fig. 4i. This data represents ~10% of all measured PD assemblies but only 0.26% of those in HC (totalling 68,569 in PD and 1,518 in HC). The existence of this bright, disease-specific shift in the nanoscale population was highly robust by ASA–PD and was observed consistently when testing different α-synuclein antibodies, two different brain banks (Queen Square Brain Bank for Neurological Disorders (QSBB) and Multiple Sclerosis and Parkinson’s Brain Bank (Imperial), 12 individuals (6 PD and 6 HC), and using different antigen retrieval methods (formic acid and heat mediated epitope retrieval) (Supplementary Note 3.3, Supplementary Figs. 15–17 and Supplementary Tables 2 and 3).

### Subpopulations of nanoscale α-synuclein assemblies are detectable by biochemical methods

Our approach enables the direct visualization of nanoscale aggregates that are typically challenging to detect. To further investigate the nanoscale assemblies revealed by our approach and determine if the fraction of bright nanoscale aggregates found in disease PD tissue could be detected with orthogonal methods, we performed PLA, enzyme-linked immunosorbent assays (ELISA), size exclusion chromatography (SEC) and seed amplification assays (SAAs) in brain tissue samples.

PLA can detect protein–protein interactions using antibody-linked DNA probes^34^. When these probes are adjacent, they produce an amplified signal that can be visualized using fluorescence microscopy (Supplementary Fig. 19a). This approach can be used to detect nanoscale assemblies of α-synuclein by amplifying the signal from α-synuclein 211 antibodies that are in close proximity. PLA was performed on 13 brain sections from the anterior cingulate gyrus, comprising six late Braak stage 5–6 PD and seven HC samples (Supplementary Table 3), and imaged (20× magnification, NA 0.75). Four images of the cingulate cortex were taken per sample and fluorescent puncta were quantified based on their intensity and a minimum size threshold of 0.9 µm. Representative images and the puncta density is shown in Supplementary Fig. 19b,c plotted per sample. The average number of puncta revealed an enriched population of aggregated α-synuclein in PD samples compared with HC samples, consistent with the ASA-PD data.

Next, brain lysates were fractionated using SEC to separate soluble α-synuclein species by apparent molecular weight from the Braak stage five or six PD, and HC donors (three PD and three HC; Supplementary Fig. 20a). This was followed by ELISA on the fractions to quantify the absolute amount of αSyn per fraction. Fractions were then subjected to the SAA, which allows the amplification and detection of small amounts of aggregates present in a sample. Recombinant human monomeric α-synuclein was used for the amplification of templated aggregation from pre-existing aggregates, and the kinetics of the amplification reaction was monitored by Thioflavin T (ThT) binding and increase in its fluorescence intensity.

The SEC-ELISA analysis revealed that total α-synuclein concentrations differed across high and low molecular weight fractions by two orders of magnitude but did not reveal statistical differences in the total α-synuclein in PD compared with controls, suggesting a similar amount of total protein (Supplementary Fig. 20b). The SAA revealed that high molecular weight fractions (200 kDa to 5 MDa) from PD brains had significantly shorter lag times, indicating the presence of seed competent αSyn aggregates, compared with samples derived from healthy brain tissue. Physiological (low molecular weight) fractions, in contrast, showed no statistical differences in lag times between PD and HC samples (Supplementary Fig. 20d,e). Comparisons between high molecular weight and low molecular weight fractions could not be made owing to altered α-synuclein concentrations. Therefore, there is a relatively small amount of aggregated α-synuclein (according to ELISA approx. 0.5% of total) both in physiological as well as disease tissue. In PD tissue, however, there is an apparent conversion of non-seed competent, or physiological, α-synuclein aggregates into seed competent aggregates.

Finally, we validated the presence of Proteinase K-resistant fluorescent puncta to assess whether the observed aggregates exhibited distinct chemicophysical properties (Supplementary Fig. 21). Notably, some bright puncta persisted after treatment, indicating a measurable degree of resistance to Proteinase K.

Collectively, the ASA–PD and amplification assay data confirm the presence of a population of small, soluble protein assemblies in the brain, which changes in PD samples, namely a subpopulation of bright assemblies has distinctive chemicophysical properties, such as their size and seed competence, and Proteinase K resistance.

### Disease-specific assemblies are spatially heterogeneous

**Fig. 5:**
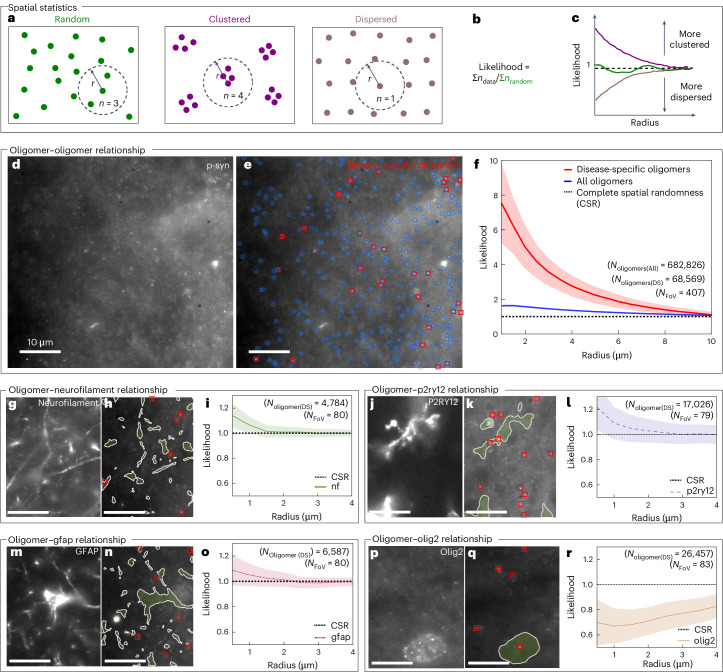
Spatial distribution of oligomers with respect to different brain cell types. **a**, An illustration showing example spot patterns with random, clustered and dispersed underlying spatial distributions. **b**, The equation used here for likelihood calculation. **c**, An example likelihood plot showing likelihood as a function of radius for random, clustered and dispersed spatial distributions. **d**, An example of an analysed FOV with an antibody stain for p-syn. **e**, The same analysed FOV with detected oligomers in blue and red, with red serving to highlight the disease-specific oligomers. **f**, A plot showing the likelihood of oligomer–oligomer distances, with 95% confidence interval presented as the shaded region, shows that all oligomers tend to spatially cluster, and that disease-specific oligomers have a higher clustering tendency. **g**–**r**, Example FOVs stained with antibodies for neurofilaments, P2RY12, GFAP and Olig2 (**g**, **j**, **m** and **p**), the same images with labelled cells shown in green and disease-specific oligomers highlighted in red (**h**, **k**, **n** and **p**) and a plot showing the likelihood of oligomer–cell distances, with the 95% confidence interval presented as the shaded region, which shows that disease-specific oligomers tend to cluster in or around neurons, microglia and astrocytes, while being dispersed from oligodendrocyte nuclei (**i**, **l**, **o** and **r**).

In addition to measuring object densities and size distributions, ASA–PD can be used to analyse spatial distributions (Fig. 5). To determine if the heterogeneity reported for larger α-synuclein aggregates^4,55^ extends to nanoscale species in PD samples, we performed a spatial-clustering test, which compares the likelihood of encountering assemblies as a function of the distance from it^53^ (Fig. 5a–c). Over 400 FoVs, 682,826 nanoscale objects were detected and characterized. On average, these species were found to cluster relative to a complete spatial random distribution. (Fig. 5f, blue); however, the disease-specific populations exhibited a substantially higher degree of clustering (68,569 aggregates; Fig. 5f, red).

ASA–PD can also be used to interrogate the distance of objects to cell-specific markers in co-stained samples. To do so, we co-stained samples with α-synuclein and various cell markers, minimizing photobleaching and optimizing the signal of the nanoscale species. We then quantified the density as a function of the proximity to the cell markers (Fig. 5g–r). For some cell types, namely neurofilament (neurons), P2RY12 (microglia) and GFAP (astrocytes), the disease-specific species showed an increased probability of being localized in proximity to neurofilament (neurons), P2RY12 (microglia) and GFAP (astrocytes). By contrast, disease specific species have a low probability of being localized to the Olig2 marker (oligodendrocyte nuclei). This approach can thus enable quantitative statistical analysis of differences of disease-specific species density over large data sets.

## Discussion

We have demonstrated the direct detection of single α-synuclein assemblies in human post-mortem brain tissue and performed quantitative analysis of more than 1.2 million objects across 30 tissue sections (682,826 nanoscale assemblies detected across 18 PD tissue sections, compared with 582,026 nanoscale assemblies detected across 18 HC sections). The acquisition of this large-scale dataset was made possible by the imaging component of ASA–PD, which is a combination of background suppression and high-NA collection of light that improves the signal-to-noise sufficiently to visualize the dim signal from individual nanoscale aggregates. The analysis pipeline that forms the detection step of ASA–PD allowed the sensitive and precise detection of these dim signals, as well as the determination of cell-specificity from co-staining and cell segmentation. The detection of small protein assemblies in post-mortem tissue is sensitive to many experimental fluctuations, and therefore ASA–PD was tested in a range of conditions, including the source of the brains (from two independent brain banks, Supplementary Table 3) and the traditional antigen retrieval methods (formic acid and heat mediated epitope retrieval). We found that the most consistent detection of oligomers was obtained using formalin-fixed paraffin-embedded (FFPE) Braak stage 6 tissue sections from a single brain bank, without any formic acid antigen retrieval; however, broadly similar trends were observed in all conditions tested (Supplementary Note 3.3 and Supplementary Fig. 15).

In agreement with the classical studies of neuropathology in PD post-mortem brains, we observed a six-fold increase in the number of aggregates above the diffraction limit in PD with respect to HC samples, and a 21-fold increase for aggregates >5 µm^2^, that is, aggregate sizes in line with Lewy pathology^4^. Lewy pathology, consisting of Lewy bodies and Lewy neurites, are microscale aggregates that are always found in sporadic PD cases (and have been used to define the disease^4,56^) and are sometimes found in HC tissue, where it has been described as incidental Lewy Body Disease^57^. Such studies have described the presence or absence of large Lewy aggregates, reporting an estimated level of abundance as a marker of severity of Lewy pathology, which lacks detailed quantitation. The technical advances implemented in ASA–PD have allowed us to capture the broad size range of protein aggregates containing phosphorylated α-synuclein and the quantification of their sizes and frequency in human PD tissue.

With this large-scale dataset, we established that there is a continuum of aggregate sizes in the human brain, ranging from large (microscale) to very small (nanoscale). Using ASA–PD, each stage of the protein aggregation pathway present in disease brain can now be measured. Importantly, there are notably fewer large aggregates compared with small aggregates, as we measure a 16-fold increase in small aggregates below the optical diffraction limit compared with larger ones in disease. Therefore, small aggregates are by far the more abundant aggregate species, underscoring the need to determine the nature of their link with PD, a long-standing unanswered question in the field. Small aggregates have been previously shown to be increased in brains with Lewy pathology compared with controls^58–60^, and elevated levels of oligomers have been detected in the cerebrospinal fluid of patients with PD compared with controls, increasing with disease severity^61–65^. Furthermore, a PLA-based approach revealed a widespread distribution of α-synuclein oligomers in disease brain tissue, in contrast to canonical microscale Lewy-related pathology^66^.

ASA–PD revealed an abundance of nanoscale assemblies in both control and PD tissue (equating to hundreds of points per cell). Their presence in control and disease suggests that small α-synuclein aggregates form under physiological conditions, where their formation and clearance are kept in balance by the protein homoeostasis system. For instance, the existence of α-synuclein as a tetramer or higher-order conformations has been previously reported in healthy-control samples using biochemical approaches^67^. Furthermore, serine 129 phosphorylation (pS129) may arise under homoeostatic conditions in response to synapse activity, being a reversible event that may regulate neuronal activity^68^. Activity-induced pS129 leads to conformational changes that facilitate interactions with new binding partners at the synapse, enabling α-synuclein to attenuate neurotransmission throughout regulating neurotransmitter release^69^. The abundance of small assemblies of phosphorylated α-synuclein in both healthy and PD brains would be in keeping with these proposed physiological roles for pS129 α-synuclein. Given the abundance of small assemblies in ASA–PD, and the known physiological role of α-synuclein, it is possible that ASA–PD is detecting α-synuclein assemblies at synapses, highlighting the potential for this approach to localize different subpopulations of assemblies in future.

The large throughput enabled by ASA–PD makes it possible to characterize the subpopulation of disease-specific species, which represent just 9.7% of the total nanoscale assemblies. The existence of a disease specific subpopulation in late-stage PD brain was supported by amplification-based methods that further verified their presence. Furthermore, characteristics of this disease-specific soluble population, in particular, their ability to exhibit prion-like activity and therefore be seed competent was demonstrated for the higher molecular weight disease specific species. Our results suggest that, in the progression of PD, a fraction of the physiological assemblies detectable in HC undergo a transition to the disease-specific species detected in PD, consistent with prior in vitro studies that identified ‘type A’ and ‘type B’ oligomers, respectively^17,19^. Once this transition has occurred, these objects, which were detected at 21-fold higher frequency than the large aggregates, may then ultimately aggregate further and become the fibrillar structures that are found in Lewy bodies and Lewy neurites. However, as ASA–PD or SAA cannot resolve the underlying structural differences between pre-fibrillar oligomers and short fibril fragments, further investigation would be needed to confirm this theory.

Nevertheless this hypothesis is consistent with previous findings of aggregation kinetics in vitro^38,70–72^, where a small proportion of the total α-synuclein population, under pro-aggregation conditions, will give rise to disease-specific, more toxic oligomers. In in vitro systems and human neurons, the earliest oligomers formed are Proteinase K sensitive, relatively inert and non-toxic; during aggregation, these oligomers undergo a transition to larger, Proteinase K resistant forms^15,17,37,73^, consistent with our observation of resistant aggregates in tissue. This transition is associated with structural conversion from relatively disordered assemblies to highly ordered and toxic species with the acquisition of β-sheet structure. It is the acquisition of this β-sheet structure that is associated with the propensity for these in vitro formed pathological oligomers to disrupt membranes and induce toxicity in human neuronal systems^74^. It is not yet clear how a structural conversion to the disease-specific species may occur in the brain. However, ASA–PD’s ability to detect a broad spectrum of aggregates including the disease-specific subpopulation opens the door to further investigate their structural properties, specifically changes in their order, sensitivity to degradation and β-sheet structure^75^.

While microscale α-synuclein inclusions have been found predominantly in neurons in PD^3,55^, less is known about smaller species. Using a spatial analysis that captures both the distances between assemblies and assemblies to cell boundaries, our data suggest that the disease-specific species cluster inside or around cell types, including neurons, astrocytes and microglia. As large (Lewy) aggregates are principally located within neurons, it is possible that the observed protein assemblies transition from their physiological state into the pathological state (either by size or by structural conversion), where they may precede the formation of the later stage Lewy bodies. How the brighter, disease-specific species ultimately form Lewy bodies containing not only fibrillar α-synuclein but also neuronal lipid membranes and organelles^76,77^, is not known in vivo. Detailed cellular and subcellular imaging of these assemblies as they transition from the bright disease-specific species to fibrillar structures are needed to provide information on the trajectory of Lewy body formation within neurons in the human brain, their heterogeneity and the components within them.

While this study has involved the analysis of over 1 million aggregates, several limitations still pose further challenges:

First, the determination of aggregate sizes: we have not directly measured the physical dimensions of the assemblies but inferred the distribution of sizes on the basis of their relative integrated brightnesses. Above the diffraction limit (around ~200 nm), we observed a strong linear relationship between the integrated brightness of aggregates and their measured area. We hypothesize that this pattern persists below the diffraction limit as well. The linear correlation with area suggests that microscale aggregates have numerous exposed epitopes on their surfaces, making it unlikely for antibodies to penetrate deeply inside the aggregates.

Second, the detection of a distinct aggregate class: we validated the use of multiple antibodies and primarily used an antibody to the serine 129 C-terminal phosphorylated form of α-synuclein. Therefore, we cannot be certain that the shifted distributions and densities observed in PD tissue are conserved for all α-synuclein assemblies. Indeed, antibodies to multiple epitopes, including those targeting the N terminus, have been shown to provide more detailed information on other aggregate subtypes and locations in the brain^78,79^.

Third, the detailed characterization of the disease-specific subpopulation: this will require super-resolution methods or electron microscopy to visualize nanoscale structures. To use super-resolution methods, further signal-to-noise ratio improvement is needed. This could include background reduction (for example, optical-clearing techniques^80^) combined with better sectioning (for example, confocal microscopy or thinner tissue slicing).

Fourth, the extended coverage of brain regions: higher throughput methods are also needed to increase statistical confidence in biological findings at the macroscopic scale of the brain. While high-throughput methods with single-molecule sensitivity are possible, there are currently no commercially available solutions, necessitating the use of custom instruments.

We acknowledge the limited sample numbers in this study but estimate from these data that an approximate two-order-of-magnitude increase in throughput of the ASA–PD method will enable the exploration of different brain regions, encompass more cases and facilitate large-scale automation, ultimately establishing a foundational aggregation map of the PD brain.

## Conclusions and outlook

We designed ASA–PD, a platform technology for the large-scale imaging of protein aggregates in brain tissue. We then demonstrated its application to generate the largest dataset so far describing the distribution of α-synuclein aggregates, their prevalence, and their spatial location in the PD brain. Without this quantitative information, it has been challenging to establish the nature of the link between α-synuclein aggregation and PD. Although substantial evidence from model systems has implicated small α-synuclein assemblies in pathological processes, it has remained unclear whether such processes are truly relevant in the disease. With ASA–PD, we identified a subpopulation of small assemblies present in disease tissue. The ASA–PD platform can thus be used to design mechanistic studies to understand how these species are created. These studies will build on the ability of ASA–PD to address the regional and cellular microenvironments that promote the development of disease-specific species, as well as the temporal evolution of the end-stage Lewy body pathology from these objects. Furthermore, integrating the ASA–PD dataset with other single-cell and spatial RNA and protein technologies will allow the identification of the key pathways and mechanisms associated with the cellular environments that promote protein aggregation and Lewy body formation. We also note that the ASA–PD method is widely applicable to other neurodegenerative diseases, where the role of protein aggregation remains largely unresolved.

## Methods

### Detailed protocols and software packages

The detailed protocols and software packages for single-molecule slides for fluorescence microscopy^81^, free-floating mouse brain immunohistochemistry^82^, preparing tissue staining and imaging^83^ and feature-detection software^84^ are available online.

### Tissue selection

Post-mortem brain tissue was obtained from Queen Square Brain Bank for Neurological Disorders (QSBB), University College London, and Multiple Sclerosis and Parkinson’s Brain Bank, Imperial College London (Imperial). Braak stage 3/4 PD cases (*n* = 3) were obtained from Imperial, Braak stage 6 PD cases (*n* = 4; 1 for technical controls, 3 for main study) from QSBB and three HCs from each brain bank to control for brain bank processing effects (*n* = 6; 3 from Imperial, 3 from QSBB) as highlighted in Supplementary Table 2. Standard diagnostic criteria were used to determine the pathological diagnosis. Case demographics for each case are described in Supplementary Table 3.

### Immunofluorescence tissue preparation

Then, 8-µm-thick FFPE tissue sections from the cingulate cortex were cut from the cases summarized in Supplementary Table 2 and described in detail in Supplementary Table 3. These sections were loaded onto Superfrost Plus microscope slides. FFPE sections were baked at 37 °C for 24 h and then 60 °C overnight. They were deparaffinized in xylene and rehydrated using graded alcohols. Endogenous peroxidase activity was blocked in 0.3% H_2_O_2_ in methanol for 10 min to suppress autofluorescence^85,86^. All sections underwent heat-mediated epitope retrieval for 10 min in citrate buffer (pH 6.0). Half of the sections were additionally incubated in formic acid for 10 min before heat-mediated epitope retrieval to test the optimal antigen retrieval conditions as these conditions are used routinely for diagnostic work and have worked previously in our experiments^87^. Non-specific binding was blocked with 10% dried milk solution in phosphate buffered saline (PBS). Tissue sections were incubated with primary antibodies anti-α-synuclein (LB509, AB_2832854 1:100; phospho S129 rabbit polyclonal, AB_2270761, 1:200; phospho S129 mouse monoclonal, AB_2819037, 1:500), anti-P2RY12 (AB_2669027, 1:100), anti-neurofilament (RT-97, AB_2941917, 1:200), anti-Glial fibrillary acidic protein (GFAP) (5C10, AB_2747779, 1:1000) and anti-Olig2 (AB_570666 1:100) for 1 h at room temperature, washed three times for five minutes in PBS followed by the corresponding AlexaFluor (anti-mouse 488, AB_2534069/anti-mouse 568, AB_144696/anti-rabbit 488, AB_143165/anti-rabbit 568, AB_143157 all at 1:200) for 1 h at room temperature. Sections were kept in the dark from this point onwards. Sections were then washed three times for 5 min in PBS and incubated in Sudan Black (multiple concentrations and incubation times were tested, as described in ‘Background suppression’ section). Sudan Black was removed with three washes of 30% ethanol before they were mounted with Vectashield PLUS (Vector Laboratories, H-1900), coverslipped (22x50 mm #1, VWR, 631-0137) and sealed with CoverGrip sealant (Biotium, 23005) for imaging. Sections were stored at 4 °C until imaging.

### Single-molecule secondary antibodies

Coverslips (24 × 50 mm, #1, VWR, 48404-453) were argon plasma cleaned (Ar plasma cleaner, PDC-002, Harrick Plasma) for 30 min before a trimmed gasket was placed on top (CultureWell™ Reusable Gasket, 6 mm diameter, Grace Bio-Labs, 103280). Poly-l-Lysine (PLL) (0.01% w/v PLL, Sigma-Aldrich, P4707) was placed in the wells for 30 min. The PLL was removed, the wells washed three times with PBS (pH 7.4, 1x Gibco, Thermo Fisher Scientific, 10010023), and the secondary antibody of choice was added (Alexa Fluor 568 goat anti-mouse—AB_144696 or Alexa Fluor 568 goat anti-rabbit—AB_143157) at a dilution of 1:10,000 in PBS from 2 mg ml^−1^ stock to a final concentration of 0.2 μg ml^−1^. The antibodies were left in the wells for 5–10 s for sufficient surface density before the wells were washed three times with PBS. PBS (30 μl) was left in the wells for imaging. Images were taken over two slides with 25 FOVs per slide.

### Microscopy

Images of human post-mortem brain and single-molecule antibodies were taken using a custom-built widefield fluorescence microscope that has been described previously^88^. Illumination of the sample was by a 488 nm laser (iBeam-SMART, Toptica) and a 561 nm laser (LaserBoxx, DPSS, Oxxius), both of which had the same excitation alignment. Both lasers were circularly polarized using quarter-wave plates, collimated and expanded to minimize field variation. The laser lines were aligned and focused on the back focal plane of the objective lens (100× Plan Apo TIRF, NA 1.49 oil-immersion, Nikon) to allow for the sample to be illuminated by a highly inclined and laminated optical sheet (HILO). Emitted fluorescence was collected by the objective lens before passing through a dichroic mirror (Di01-R405/488/561/635, Semrock). The collected fluorescence then passed through emission filters dependent on the excitation wavelength (FF01-520/44-25 + BLP01-488R for 488 nm excitation, LP02-568RS-25 + FF01-587/35-25 for 561 nm excitation, Semrock). The fluorescence was then expanded (1.5×) during projection onto an electron-multiplying charge-coupled device (EMCCD, Evolve 512 Delta, Photometrics). The EMCCD was operating in frame transfer mode with an electron multiplication gain of 250 analogue-to-digital units per photon.

Z-stacks of images were taken through the samples in 0.5 µm steps with 17 steps per FoV covering a depth of 8 µm. To reduce bias in FoV selection, nine FoVs were recorded in a grid formation of 3 × 3 FoVs with 150 µm spacing, implemented with the ImageJ2^89^ Micromanager plugin^90^. Three 3 × 3 grids were recorded at three random locations within the grey matter of each section, resulting in 27 z-stacks per section. Images were recorded with 1 s exposure time of the EMCCD. Images were recorded with a power density of 2.4 W cm^−2^ (488 nm excitation) and 25.9 W cm^−2^ (561 nm excitation).

### Camera calibration

To convert the pixel values from counts in analogue-to-digital units to photons, a series of images were recorded at different illumination intensities, including one taken under no light to measure the camera (EMCCD, Photometrics, Evolve 512 Delta) offset^91^. Each intensity was captured in 500 frames, resulting in a total of 3,000 frames across the six different illumination levels. For every pixel, the mean and variance was calculated across the 500 frames, generating six different variance and mean values corresponding to the six illumination intensities. The dark counts, that is, the camera offset per pixel, were determined as the mean pixel value in the dark frame. The camera gain per pixel, expressed in photoelectrons per count, was determined by calculating the slope between the six variance and mean values per pixel, subtracting the dark frame offset.

### Feature detection

Custom code was written to detect both cells and α-synuclein aggregates. Firstly, each z-stack was manually inspected to remove out-of-focus images. For the aggregate channel, two binary masks representing nanoscale and larger fluorescent objects images respectively were created for each image using the following four steps: (1) the positions of large objects were detected with a difference-of-Gaussian kernel (*σ*_1_ = 2 px, *σ*_2_ = 40 px) and Otsu’s threshold, (2) cellular autofluorescence was removed using high-frequency filtering (*σ*_high-pass_ = 5 px), and diffraction-limited-sized features were enhanced through Ricker wavelet filtering (*σ*_Ricker_ = 1.1 px); (3) bright spots within the image were identified using an intensity threshold on ther basis of the top 2.5th percentile of the image; and (4) spurious pixels were removed from the union of the two binary masks in step 1 and 3 via morphological opening operation with a disk shape (radius of 1 px). The resulting mask is then separated into nanoscale and non-nanoscale fluorescent objects, where the threshold was 19 px, determined by 40 nm subdiffraction limited fluorescent beads (40 nm, FluoSphere F10720). For the cell channel, cell segmentation was achieved using the function described in the first step of the aggregate-detection pipeline but using different kernel sizes (*σ*) dependent on cell type. For specific details of *σ* values used, see Supplementary Information Note 3.1. All imaging data were viewed in ImageJ and analysed and plotted using custom MATLAB code and Origin.

### Disease-specific definition

The disease-specific subpopulation was defined relative to the corresponding HC datasets after outlier removal on the basis of 1.5 times the IQR. This corresponds to the ~99.7 brightness percentile of the nanoscale assemblies identified in HC patients.

### Reporting summary

Further information on research design is available in the Nature Portfolio Reporting Summary linked to this article.

## Supplementary information

Supplementary Information Supplementary Figs. 1–20, Discussion and Tables 1–6.

Reporting Summary

## Data Availability

All unprocessed data^35^, processed data (binary masks, density and intensity information)^36^, methods and detailed protocols are available online.

## Code availability

Analysis code is available online.

## Declarations

Ethical approval for the study was granted by the Local Research Ethics committee of the National Hospital for Neurology and Neurosurgery.
