# Supplementary material for: Large-scale visualization of α-synuclein oligomers in Parkinson’s disease brain tissue
Source: Nat Biomed Eng. 2025 Oct 1;10(6):1222–34. doi: 10.1038/s41551-025-01496-4 (PMC13278957; doi:10.1038/s41551-025-01496-4)
Supplement: Supplementary file 1 — Supplementary Figs. 1–20, Discussion and Tables 1–6. [file 41551_2025_1496_MOESM1_ESM.pdf]

nature biomedical engineering

Supplementary information https://doi.org/10.1038/s41551-025-01496-4

Large-scale visualization of α-synuclein oligomers in Parkinson's disease brain tissue

In the format provided by the authors and unedited

Table of Contents

1. Supplementary Methods
2. Supporting Figures (Supplementary Figures S1-14)
3. Supplementary Notes (containing Supplementary Figures S15-20)

   3.1: Aggregate-detection pipeline

   3.2: Simulations

   3.3: Detailed sample-preparation protocols

   3.4: Properties of ASA-PD in comparison to orthogonal aggregate detection methods
4. Object density with segmented cells
5. Supplementary Tables S1-6
6. Supplementary References

1. Supplementary Methods

Transgenic mouse models:

We used *SNCA*-OVX mice (n = 4; B6.Cg-Tg[SNCA]OVX37Rwm *Snca*^{tm1Rol}/J; Strain #023837; The Jackson Laboratory) and *Snca* KO mice (n = 4; B6;129X1- *Snca*^{tm1Rol}/J; Strain #003692; The Jackson Laboratory), aged 3.5-4.0 months old**.** Both male mice (n = 6) and female mice (n = 2) were used. Sex was assigned on visual inspection of mice by trained staff. Sex was not considered in the study design. Sex-based analyses were not performed because the small sample sizes did not provide sufficient power for rigorous testing. *SNCA*-OVX mice and *Snca* KO mice used in this study were littermates, generated by breeding *SNCA*-OVX mice with *Snca* KO mice. All mice were group housed after weaning (according to sex) in individual ventilated cages with environmental enrichment, had *ad libitum* access to food and water, and were maintained at 20-24 ºC and on a 12:12 hour light:dark cycle. Zeitgeber time (ZT; with ZT0 = lights on in the animal facility) of end-of-experiment protocol was ZT3–ZT6. This study was carried out in accordance with the Animals (Scientific Procedures) Act, 1986 (United Kingdom), under the authority of UK Home Office Project Licences PB9E6B4B1 and PP4733840.

SNCA extraction from brain materials:

Protocol was adapted from Peng 2018[92]. Samples were mechanically homogenised in HS buffer (50mM TRIS, 750mMNaCl, 5mMEDTA,10mM NaF) at pH 7.4 supplemented with protease inhibitors. Samples were then spun at 4°C and 100kg for 30 min. Supernatant was collected as the soluble fraction and loaded onto a calibrated Size Exclusion Chromatography (SEC) column. Fractions of interest were stored at -80°C.

Seed Amplification Assay (SAA) protocol:

We monitored and compared prion activity from PD patients and Healthy controls by performing an α-synuclein aggregation assay. We monitored formation of fibrils with Thioflavin T, a fluorescent dye that undergoes an excitation/emissions spectra red shift when bound to aggregates. All samples were assayed in triplicates and plates were repeated once. Samples were kept on ice and sonicated. The reaction buffer was 25mM Phosphate buffer, 170mM NaCl, 10uM ThT and a glass bead. The plate reader was incubated at 42°C and plates were shaken in-between fluorescent readings. The data was analysed by determining the lag time of the reactions by marking the time when fluorescence rose beyond 10 standard deviations of the blank control. These lagtimes were binned according to molecular weight and controls were compared to patient samples with a two tailed unpaired t-test.

Enzyme Linked Immunosorbent Assay (ELISA):

Fresh frozen grey matter from 3/4 Parkinson's disease patients and healthy controls was chipped (around 500mg total) and the samples were homogenized using overhead stirrer at 2500 rpm in 1mL cold pH 7.4 HS buffer (50mM Tris-HCl, 750 mM NaCl, 5mM EDTA, 10mM NaF, Protease inhibitors (cOmplete Mini, EDTA-free Protease Inhibitor Cocktail, 11836153001, Roche)). Homogenate was centrifuged at 4°C, 100,000g for 30 min, and the supernatant was collected as the soluble fraction. For the insoluble fraction, the pellet went through series of resuspensions and centrifugation cycles, first in HS buffer, then in HS buffer and 1% Triton X and then 3x in HS buffer with 1% Triton X and 30% sucrose (S7903 Sigma), after which the supernatant was discarded to assure absence of membranes and myelin. Then, the pellets were resuspended in 1mL of HS + 1% sarkosyl (L9150, Sigma) using a handheld homogenizer and allowed to rotate on a carousel overnight at 4°C, after which they were centrifuged and supernatant discarded. Pellets were washed in 1mL of PBS and after centrifugation and supernatant removal, the pellets were sonicated for 1min using a horn sonicator in 200ul of PBS. Sonicated samples were centrifuged at 186,000g for 20 minutes at 4°C and the supernatant was collected as the insoluble fraction. To determine the levels of α-synuclein assemblies the Human α-synuclein oligomer (non-A4 component of amyloid precursor) ELISA kit (CSB-E18033h, CUSABIO) according to the manufacturer's instructions. All standards and fractions were run in technical duplicate. The levels of α-synuclein assemblies were calculated to a four-parameter logistic standard curve and were normalized to the total protein concentration of each fraction.

Proteinase K digestion

The same tissue preparation was used as described in the methods section (Immunofluorescence tissue preparation) except for an additional incubation step before blocking using a milk solution. This additional incubation step consisted of incubation in 50 ug/uL Proteinase K solutions for 10 seconds, 1 minute or 2 minutes. Sections were then treated using a 0.1% concentration of Sudan Black for 10 minutes as described in the methods. Proteinase K treated tissue was then imaged using the spinning-disk confocal microscope that has been described previously[51].

Proximity Ligation Assay

Paraffin embedded human tissue was dewaxed by incubating sections for 2 minutes in Xylene substitute (Labbox) and then rehydrating them with a decreasing ethanol gradient. Sections were heat retrieved in a 700

W microwave in citrate buffer pH 6. They were then blocked for PLA using Navinci Reagents as instructed by the manufacturer. A-syn 211 conjugated antibody (Abcam) was used as a detection probe at 1:2000, and ligation and polymerization times were extended to 1 h and 2,5 h respectively. Sections were counterstained with DAPI (Avantor) at the end of the protocol and coverslipped with Fluorsave (Merck). Images were acquired with an EVOS M7000 and puncta were counted with ImageJ.

Antibody Evaluation and Performance Across Spatial Scales

The choice of antibody is critical for ASA-PD, as our findings reveal size-specific differences that make some commercial antibodies more suited to this method. This is influenced by a combination of specificity and affinity. Antibodies with high nonspecific binding can still generate high-contrast signals for larger aggregates at high concentrations, but this comes at the cost of increased nonspecific background that raises the noise floor masking dimmer fluorescent puncta. Conversely, using high specificity antibodies may lead to an increase in false positive fluorescent puncta, which are filtered out using our analysis pipeline[51,84]. Another important factor is the relative concentration of protein aggregates, which must be sparse enough to ensure that individual puncta are sufficiently isolated to see by diffraction-limited imaging.

Using quantitative image analysis, we compared an array of commercial antibodies targeting different epitopes of α-synuclein (Supplementary Figure S3g). Generally, ASA-PD allows visualization and quantification of large, disease-associated pathologies for many α-synuclein antibodies. Consistently showing the prevalence of these species in Parkinson's disease (PD) cases than in healthy controls (HC). For very small assemblies, e.g., oligomers or nanoscale fibrils, we found the selection antibodies to be crucial. Specifically, antibodies must be chosen to minimize background, as the higher resulting noise obscured the smaller aggregates preventing detection.

For ASA-PD to reliably detect single aggregates (<200 nm), two essential criteria must be met (Supplementary Figure S3h):

1. The total background fluorescence must not exceed **1.2×** the background fluorescence intensity of the negative control sample used for calibration.
2. Diffraction-limited puncta must be **sufficiently sparse** to ensure spatial resolution.

These findings highlight the importance of selecting antibodies that balance specificity, affinity, and background fluorescence for optimal ASA-PD performance across different spatial scales of α-synuclein pathology.

2. Supporting Figures

Autofluorescence suppression with Sudan Black B

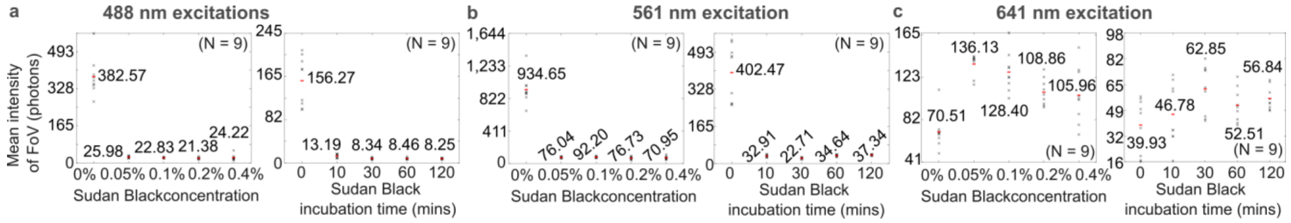

Supplementary Fig. S1 Sudan Black (SB) concentration study and incubation time study. a. Data taken at 488 nm excitation, 100 ms, 2.4 W/cm^2. **b.** Data taken at 561 nm excitation, 1 s, 25.9 W/cm^2. **c.** Data taken at 641 nm excitation, 100 ms, 7.0 W/cm^2. Black 'X' are an average of 20 FoVs, in 3 different places of grey matter, over 3 sections each from a different Braak Stage 6 PD patient (9 data points per condition). Red is the mean of all 'X' for conditions labelled with mean intensity (photons).

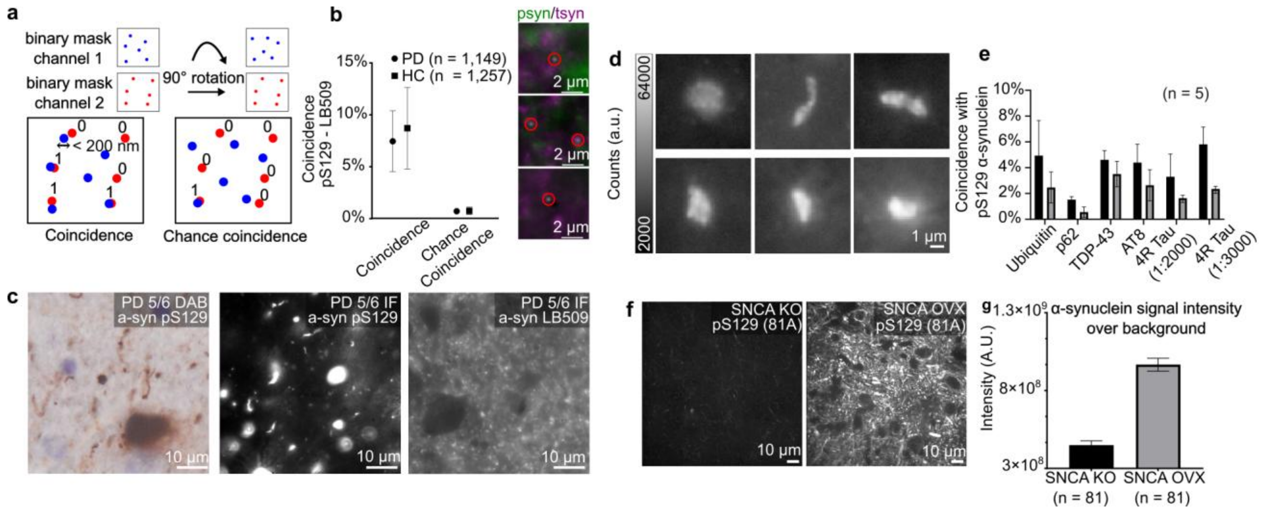

Supplementary Fig. S2 Robust α-synuclein detection with different antibodies. a. Schematic representation of the assertion of coincidence of two image channels for diffraction-limited data. Coincidence between both channels is asserted based on an overlay of two binary masks. If $n > 1$ pixel overlaps, a coincidence is registered. Chance coincidence is calculated in the same way after rotating one binary mask 90 degrees. **b.** Coincidence and chance coincidence of LB509 (AB_2832854) on pS129 α-synuclein (AB_2270761) across n = 3 Parkinson's disease Braak stage 5/6 and n = 3 HCs. The *n* in the legend represents the number of fields of view. Error bars are standard error of the mean. Images illustrate examples of pSyn-total-syn colocalization. **c.** Representative DAB-stained and two immunofluorescent images of a late-stage PD patient using the pS129 α-synuclein antibody (AB_2819037) for images 1 & 2 and a total α-synuclein antibody LB509 (AB_2832854) for image 3 showing comparable Lewy pathology produced by the pS129 antibody and increased background in immunofluorescence produced by the total α-synuclein antibody. **d.** Detailed aggregate compilation from a late-stage PD patient in immunofluorescence using the pS129 α-synuclein antibody (AB_2819037) showcasing characteristic Lewy pathology. **e.** Diffraction-limited aggregate coincidence vs. chance coincidence between pS129 α-synuclein (AB_2270761) and disease-related proteins Ubiqutin (AB_2315524), p62 (AB_398152), TDP-43 (AB_425904), AT8 (AB_223647) and 4R Tau (AB_310014) in a late-stage PD patient (n= 5 FOVs). **f.** Representative images of Mouse brain tissue imaged on a spinning-disc confocal microscope of SNCA knockout (n = 4; B6;129X1- *Snca^{tm1Rol}*/J; Strain #003692; The Jackson Laboratory) and human SNCA overexpressing mice (n = 4; B6.Cg-Tg[SNCA]OVX37Rwm *Snca^{tm1Rol}*/J; Strain #023837; The Jackson Laboratory) stained with pS129 antibody (AB_2819037), respectively. **g.** Averaged stack (132 µm x 132 µm x 12 µm) whole-image intensity of mouse brain tissue stained with pS129 antibody (AB_2819037) in SNCA KO mice not expressing α-synuclein and SNCA OVX mice overexpressing human α-synuclein across n = 3 biological replicates per genotype. Background counts from controls lacking secondary antibodies were subtracted to quantify the overall change in staining.

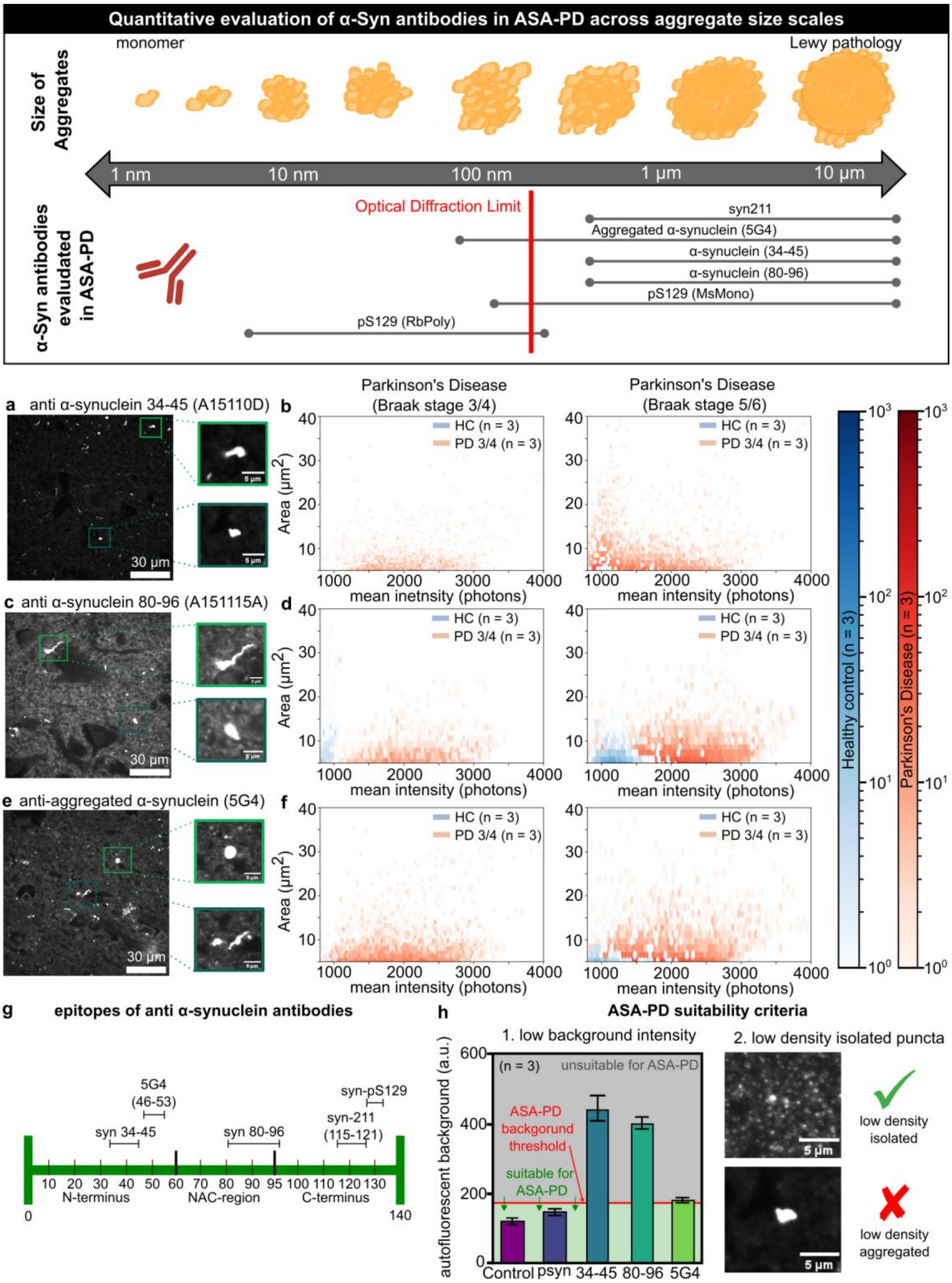

Supplementary Fig. S3: Commercial antibodies exhibit varying levels of performance across different spatial scales of protein assemblies. **a,c,e**. Example images showing tissue from the Anterior Cingulate Cortex, Braak stage 5/6 stained with (a**)** anti α-synuclein 34-45 (A15110D), (c) anti α-synuclein 80-96 (A151115A) and (e**)** anti-aggregated α-synuclein (5G4). All antibodies reveal large (>5 µm) α-synuclein aggregates. **b,d,f**. show 2D histograms of mean intensity of large (> 5 µm) aggregates vs size, for both Braak stage 3/4, 5/6 (red) and HC (blue), for the corresponding antibodies in (a,c,e). **g.** The target region for the binding epitopes. **h.** Illustration of the ASA-PD requirement for two essential criteria to be met for single aggregate detection (objects <200 nm). The *total background fluorescence* cannot exceed 1.2x the background fluorescence intensity of the negative control sample used for calibration. Secondly, diffraction-limited puncta need to be at sufficiently low enough density to be resolved spatially.

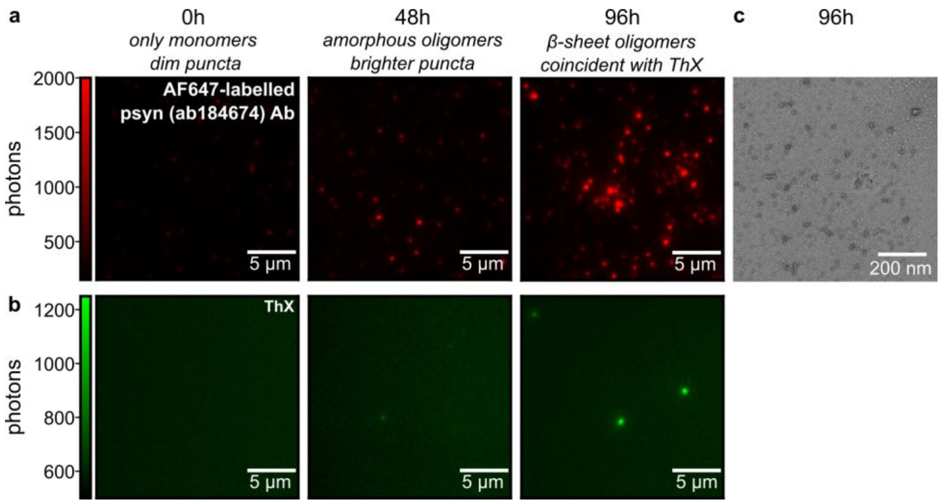

Supplementary Fig. S4: Recombinant 100% phosphorylated α-synuclein forms 10 nm assemblies *in vitro*. Recombinant phosphorylated α-Synuclein forms 10 nm assemblies in vitro. Recombinant α-Synuclein, post-translationally modified at S129, forms ~10 nm aggregates in diameter after 96h of incubation, **a-b**. shown both in fluorescence and **c.** TEM imaging. These 96h-aggregates contain β-sheet, shown by simultaneous SAVE[70] imaging. Aggregation was performed by shaking a 25 µM concentration of post-translationally modified α-Synuclein in PBS at 37°C. These objects were imaged using a customized widefield microscope[51] with 638 nm excitation for (a) AF647 labelled mouse monoclonal antibodies to p-syn (AB_2819037) (power density: 60 W/cm^2) and (b) 488 nm excitation in the case of ThX (power density: 173 W/cm^2).

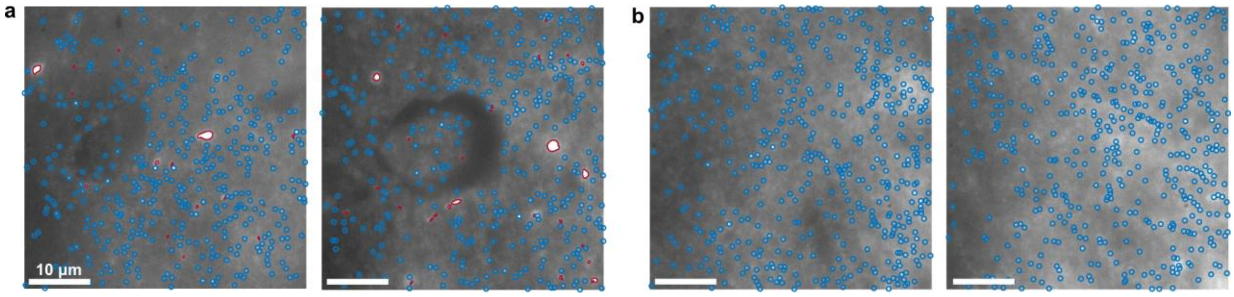

Supplementary Fig. S5 α-synuclein detection and quantification in HC and PD samples. a. Representative images of two PD patients stained for pS129 α-synuclein (AB_2270761) showing abundant diffraction-limited puncta. **b.** Representative images of two HCs stained for pS129 α-synuclein (AB_2270761) showing a comparable number of diffraction-limited aggregates detected.

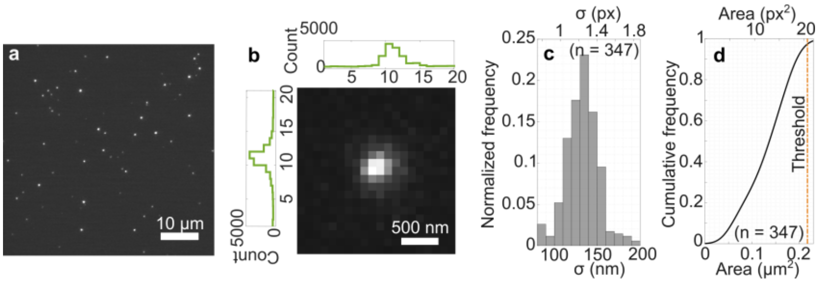

Supplementary Fig. S6 Calibration of the point-spread function with 20 nm fluorescent beads. a. Sparse TetraSpeck beads (T7279) were attached to a coverslip with poly-L-lysine and imaged with PBS using 50 mW laser power. **b.** Zoom-in of a single bead with its horizontal and vertical intensity profile. **c.** The corresponding histogram of the width parameter, σ, is extracted by localising each object with a 2D symmetric Gaussian function. **d.** Cumulative frequency of spot sizes was derived using the aggregate detection code with the same parameter used for the human brain data. The cutoff threshold for small aggregates is highlighted in orange.

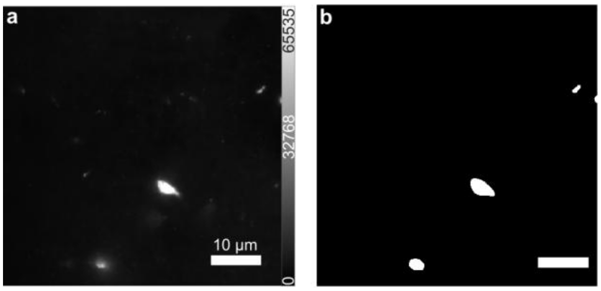

Supplementary Fig. S7 Forming a large-object binary mask. a. Raw image of a PD sample in the Anterior Cingulate Cortex stained for pS129 α-synuclein (AB_2270761). **b.** Binary mask resulting from the large object detection code.

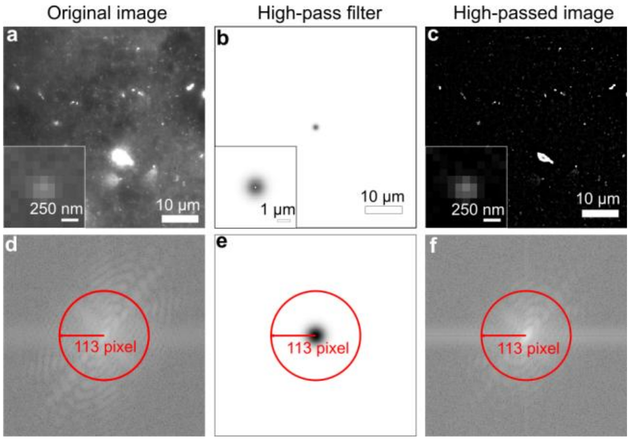

Supplementary Fig. S8 Background removal for nanoscale object detection. a-c. The magnitude of frequency domain of original image, high-pass filter and high-passed image respectively with a highlighted red circle showing the passband due to diffraction limit. **d-f.** The spatial (*i.e.* image. domain of the original image, high-pass filter and high-passed image, respectively). **(a&c)** are shown in log scale.

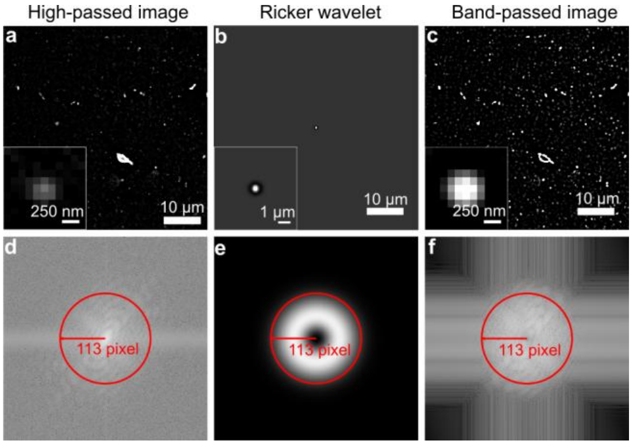

Supplementary Fig. S9 Feature enhancement for nanoscale object detection. a-c. The magnitude of frequency domain of high-passed image, ricker wavelet and band-passed image respectively with a highlighted red circle showing the passband due to diffraction limit. **d.**, **e.**, **f.** The spatial domain of high-passed image, ricker wavelet and band-passed image, respectively. **a.** and **c.** are shown in log scale.

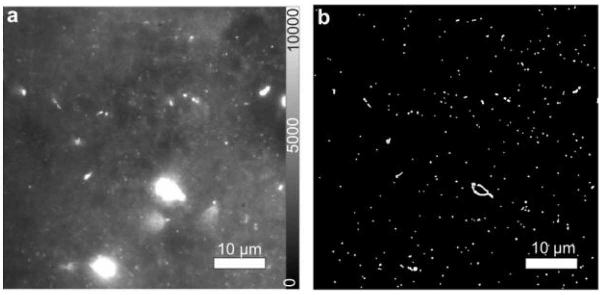

Supplementary Fig. S10 Forming a small-object binary mask. a. Original image from the anterior cingulate cortex of a PD sample stained with anti-alpha-synuclein pS129 (AB_2819037) visualised with (AB_144696). **b.** Resulting binary mask after a top 2.5 percentile thresholding.

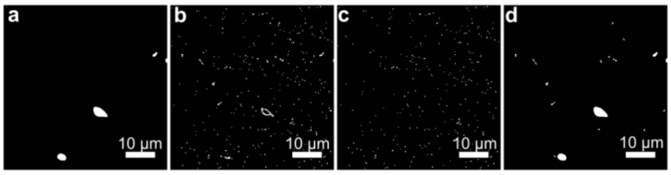

Supplementary Fig. S11 Filtering and classification output. a. Binary mask from large object detection section. **b.** Binary mask from small object detection session. **c.** Result binary mask for large objects **d.** Resulting binary mask for objects classified as diffraction-limited assemblies.

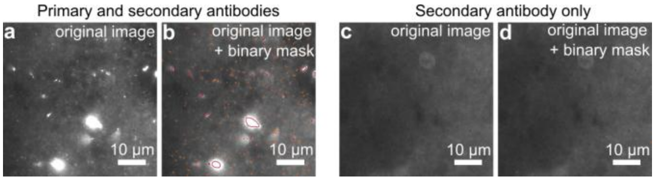

Supplementary Fig. S12 Aggregate-detection output. a.b. The original image and resulting diffraction limit and non-diffraction limit mask from a PD patient with the primary antibody targeting pS129 alpha-synuclein (AB_2270761) and the secondary Goat anti-mouse AF568 (AB_144696). **c.d.** The original image and resulting diffraction limit and non-diffraction limit mask from a PD patient stained with no primary but only the secondary antibody.

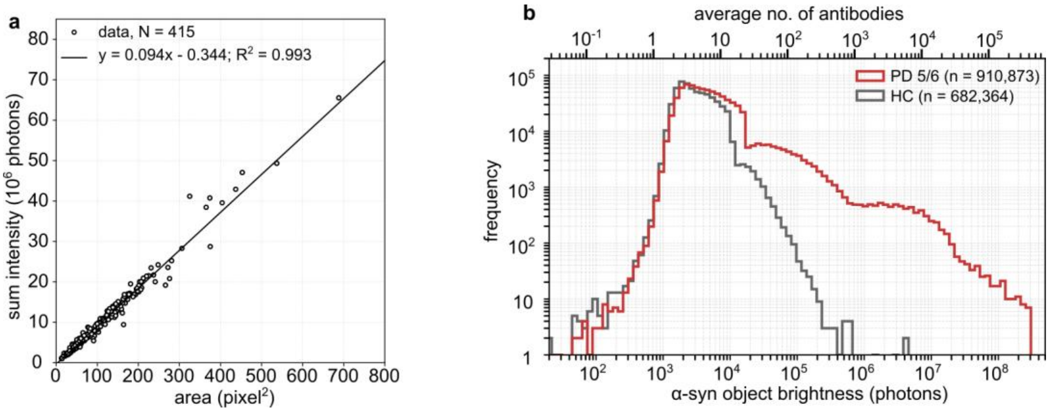

Supplementary Fig. S13 Aggregate brightnesses and size. a. Distributions of aggregate brightnesses (photons) plotted against aggregate size (pixels) for $N = 415$ aggregates above the diffraction limit. A linear fit with $R^2 = 0.993$. **b.** Frequency distribution of the average number of antibodies and the pS129 α-synuclein (AB_2819037) object brightness (photons) for $n = 910{,}873$ objects detected in PD patients, $n = 682{,}364$ objects detected in HCs.

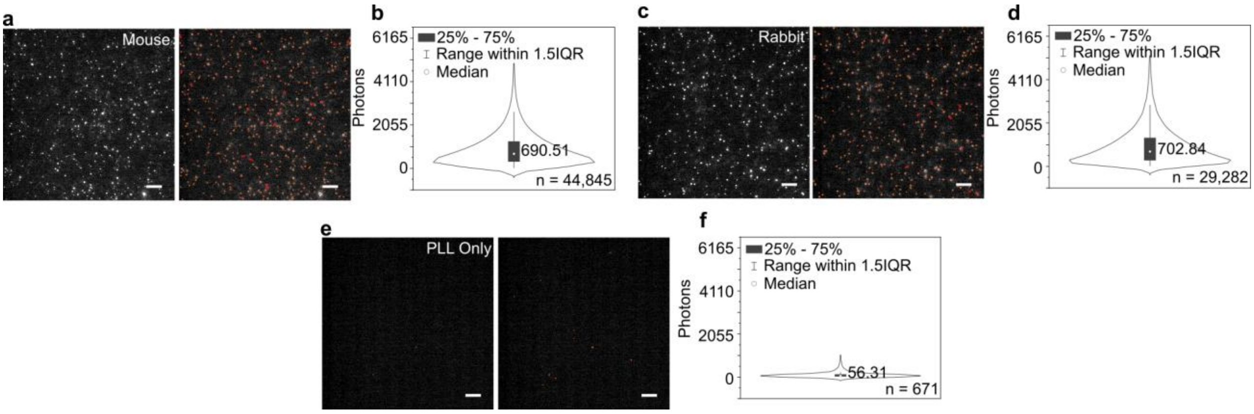

Supplementary Fig. S14: Single-molecule analysis of secondary antibodies. Secondary antibodies (AB_143157, AB_144696) were fixed on a glass coverslip and 50 FoVs across two slides were imaged for each antibody. All imaging conditions were the same as for the main brain imaging. **a,c,e.** Representative raw images and detection of spots. **b,d,f.** quantified distribution of photon numbers per localization corresponding to (a,c,e). **a,b.** shows Alexa Fluor 568 goat anti-mouse (AB_144196); **c,d.** Alexa Fluor 568 goat anti-rabbit (AB_143157). **e,f.** show a negative control sample (Poly-L-lysine and brightnesses of detected spots on pure PLL sample, i.e. contamination. Since both Alexa Fluor 568 antibodies had similar brightnesses, 700 photons/antibody was used for the approximate conversion between brightness and number of secondaries in *Supplemental Figure S13*.

3. Supplementary Notes

3.1 Simulations

In the following section, we describe the modelling assumptions and calculations done to simulate the expected effective signal-to-noise ratio of imaging single bright objects in a noisy tissue background.

We model the expected numbers of photons from a signal object and from a fixed concentration of background objects, emitting in the illuminated volume of one pixel. The pixel axial length was assumed as a depth of field is

$$DoF = \frac{\lambda n}{NA^2} + \frac{e}{M \times NA} \qquad \text{SI Equation S1}$$

where λ is the wavelength of excitation light (561 nm here), *n* the refractive index of the medium (assumed 1.515), NA is the numerical aperture, *e* is the smallest resolvable object on the camera plane (11 µm here) and M is the magnification (assumed 100 here).

We assume one bright single object that emits in this pixel, and background objects at a concentration of 100 µM (the number in the pixel will vary, dependent on the NA-dependent depth of field). We assume each background object has a photon emission rate of 122 photons s-1—this number was chosen to agree with experiment in this work. We assume that the signal objects may have one of two brightness levels, dependent on if they are "large objects" or "small objects" observed in experiment. For small objects we assume a photon emission rate of 13,000–21,000 photons s-1 and for large objects we assume a photon emission rate of between 24,000–300,000 photons s-1. Again, we emphasise that these values were chosen to agree with experiment.

Thus, the number of photons arriving from our single signal object is set, and the number of background photons is calculated in a numerical-aperture dependent manner by multiplying the volume (DoF*107^2 nm^2) by the concentration of background objects.

We then assume that the numerical aperture and the camera quantum efficiency will cause a loss of photon detection efficiency, and therefore a loss in the number of detected photons. The numerical aperture will cause a loss of efficiency proportional to the cone angle of collected fluorescence, Supplemental Equation S2.

$$Loss = QY \times \frac{1 - \cos\theta}{2} \qquad \text{SI Equation S2}$$

where the angle, θ, is calculated from the numerical aperture by Supplemental Equation S3.

$$\theta = \sin^{-1}\left(\frac{NA}{n}\right) \qquad \text{SI Equation S3}$$

Assuming a fixed numerical aperture, we calculate the signal-to-noise ratio by measuring the number of signal photons detected in 100 ms, number of background photons detected in 100 ms, as in Moerner and Fromm[93].

3.2 Aggregate-detection pipeline

Characterisation of system point spread function

It is important to distinguish nanoscale objects (e.g. oligomers and nanoscale fibrils), whose appearance on a detector is blurred by the diffraction of light, from objects larger than the diffraction limit (*i.e.* medium-sized aggregates, LBs and LNs). Even though fluorescently labelled nanoscale emitters appear approximately identical, slight variations exist due to out-of-focus emitters, optical aberrations, background variance and other sources of noise (*e.g.* shot noise and read noise). To characterise the measured area for sub-diffraction objects, we analysed images of beads (40nm, FluoSphere F10720) with our pipeline (Supplementary Figure S6). As very few beads are defocused and/or aggregated (which could produce objects larger than the diffraction limit), the area threshold is selected to include

97% of all data. Notably, closely spaced, but independent fluorescent species could appear as a single bright spot or an extended spot depending on their precise distance relative to the diffraction limit, however, this is rare at low densities. Thus, the size threshold determined from the 40nm beads was applied to classify the detected objects into punctate signal and larger species.

Step1 - Large object detection

The hallmark of Parkinson's disease is the presence of large aggregates of α-synuclein such as Lewy Bodies and Lewy Neurites. These comparatively very bright objects mask the presence of much dimmer nanoscale objects, such as oligomers, and it is therefore necessary to exclude regions of the image containing these and other large features. To do so, a large-object mask is made using a difference-of-Gaussians kernel, *i.e.* a band-pass in the frequency domain to eliminate both the background and small objects (Supplementary Equation S4, where σ_1 is 2 pixels, slightly larger than σ of the diffraction limit, and σ_2 uses 40 pixels, larger than most of LBs and LNs thus eliminating the background as much as possible). Where the large objects to be detected are instead cell types, the σ values for each cell type are **microglia**: σ_1 = 2 pixels, σ_2 = 10 pixels; **neurons & astrocyte**: σ_1 = 0.5 pixels, σ_2 = 5 pixels; **oligodendrocyte**: σ_1 = 4 pixels, σ_2 = 40 pixels.

$$h_{DoG} = \frac{1}{2\pi\sigma_1^2} e^{-\frac{x^2+y^2}{2\sigma_1 2}} - \frac{1}{2\pi\sigma_2^2} e^{-\frac{x^2+y^2}{2\sigma_2 2}} \qquad \text{Equation S4}$$

Since the area of LBs is large compared to the pixel size, a two-class Otsu's threshold[94], which separates pixels into two classes, is then applied. The resulting binary classification on an example image is shown in Supplementary Figure S7.

Step2 - Image filtering

Two different frequency-filtering steps are applied to remove the background and emphasise the diffraction-limited spots in the image, *i.e.* the nanoscale assemblies. In this dataset, the background due to autofluorescence of the brain tissue was relatively smooth. It can therefore be regarded as a low-frequency signal suppressible with a high-pass filter. This was implemented by subtracting the low-pass filtered image from the original image. Supplementary Equation S5 describes the spatial representation of the high-pass kernel, where δ is the Dirac pulse at (x,y) = (0,0), σ = 5 pixel larger than σ from sub-diffraction beads (1.3 pixel), and designed to maintain small and intermediate size aggregates that were the focus of our study. The original image, high-pass kernel and high-passed image are shown in Supplementary Figure S8.

$$h_1 = \delta(0,0) - \frac{1}{2\pi\sigma^2} e^{-\frac{x^2+y^2}{2\sigma^2}} \qquad \text{SI Equation S5}$$

After removing the low-frequency autofluorescence background, high-frequency pixel noise hinders object detection. To improve the detectability of these small objects, a feature enhancement filter, namely the Ricker wavelet is used[52]. This step acts as a band-pass filter. The wavelet, described in Supplementary Equation S6 uses σ = 1 pixel such that it is slightly smaller than the σ = 1.3 pixel from sub-diffraction beads to maximise the suppression of pixel noise. Supplementary Figure S9 shows the spatial and frequency representation of the wavelet and band-pass image, respectively. The overall formula is described in Supplementary Equation S7, where H_1 and H_2 represents the Fourier transform of h_1 and h_2 and i_{init} and $i_{filtered}$ represents the image in the spatial domain. The system bandwidth, calculated in pixels in Supplemental Equation S8 as the Rayleigh diffraction limit in the frequency domain, is shown in Supplementary Figures S8 and S9 as the red circle.

$$\frac{1}{\pi\sigma^4}\left[1 - \frac{1}{2}\left(\frac{x^2+y^2}{\sigma^2}\right)\right] e^{-\frac{x^2+y^2}{2\sigma^2}} \qquad \text{SI Equation S6}$$

$$i_{bandpass} = \mathcal{F}^{-1}\{\mathcal{F}\{i_{init}\} \times H_1\{x,y;\sigma\} \times H_2\{x,y;\sigma\}\} \qquad \text{SI Equation S7}$$

$$f_{rayleigh} = \frac{NA}{0.61\lambda} \times Pixelsize \times Image\ width\ = 113 pixel \qquad \text{SI Equation S8}$$

Step3 - Image thresholding

After background and autofluorescence suppression, a threshold is used to identify potential regions of interest within the image. Due to the variability in the number of diffraction-limited spots visible during imaging (typically between ~30–300), a threshold value of top 2.5 percentile of the filtered image value was used. This value was determined experimentally based on the relatively small percentage of pixels containing nanoscale assemblies, while avoiding the influence of a small number of very bright objects that sometimes remained after the previous filtering steps. The resulting binary mask is shown in Supplementary Figure S10, denoted as $i_{threshold}$.

Step 4 - Object filtering and classification

Due to the diffraction limit of light, nanoscale fluorescent objects are diffraction-limited and appear to have similar shapes. A morphology filter is used to reduce the number of false positives due to spuriously bright pixels. Specifically, an opening filter is applied by first eroding and then dilating the binary image with a disk shape with a diameter of 3 pixels. Furthermore, there are also false positives from LBs in the FoV, which can be eliminated by the known position from the large object detection section. Supplementary Equation S9 represents the object filtering process where i_{lb} is the binary mask from the large object detection and B is a pre-defined binary structure, specifically a binary disk with radius 1. This effectively compares every object in the image with the disk structure, and everything smaller than the disk or dissimilar to the disk will be erased. Further details can be found in Haralick et al.[95]. Objects in the binary mask i_{bw} are then classified into nanoscale assemblies and larger objects separately.

$$i_{bw} = i_{opening} \cup i_{lb}\ where\ i_{opening} = (i_{threshold} \ominus B) \oplus B \qquad \text{SI Equation S9}$$

3.3: Detailed sample-preparation protocols

The protocol employed for sample preparation stemmed from an established protocol for antigen retrieval at the Queen Square Brain Bank. To determine how generalizable the protocol is and how it might affect the appearance of samples by high-resolution microscopy, we compared sources of the brain tissue (brain bank), fixation state of the tissue (FFPE vs fresh-frozen), and antigen retrieval methods (In FFPE, formic acid and heat-mediated epitope retrieval vs just heat-mediated epitope retrieval), and autofluorescence suppressor concentrations. The same three HC and PD cases were used for these comparisons throughout (PD cases: 8, 9, 10 and HC cases: 11, 12, 13, Supplementary Table 3).

Imperial brain bank vs Queen Square Brain Bank:

We found that the brightness of assemblies detected differed between the two brain banks. This meant we needed to always include controls matched to the brain bank the PD cases came from (Supplementary Figure S15a,b).

Formic acid and heat-mediated epitope retrieval vs only heat-mediated epitope retrieval on FFPE tissue:

We found that both antigen retrieval methods produced similar results (Supplementary Figure S15d) which resulted in us going forward with only heat-mediated epitope retrieval for ASA-PD analysis. These measurements were highly repeatable across multiple tissue patients of the same patient (an example shown in Supplementary Figure S16).

FFPE vs Fresh-frozen:

We found that FFPE samples more consistently detected the bright disease-specific species when compared to frozen samples (data not shown) which concluded in us going forward with FFPE for ASA-PD analysis.

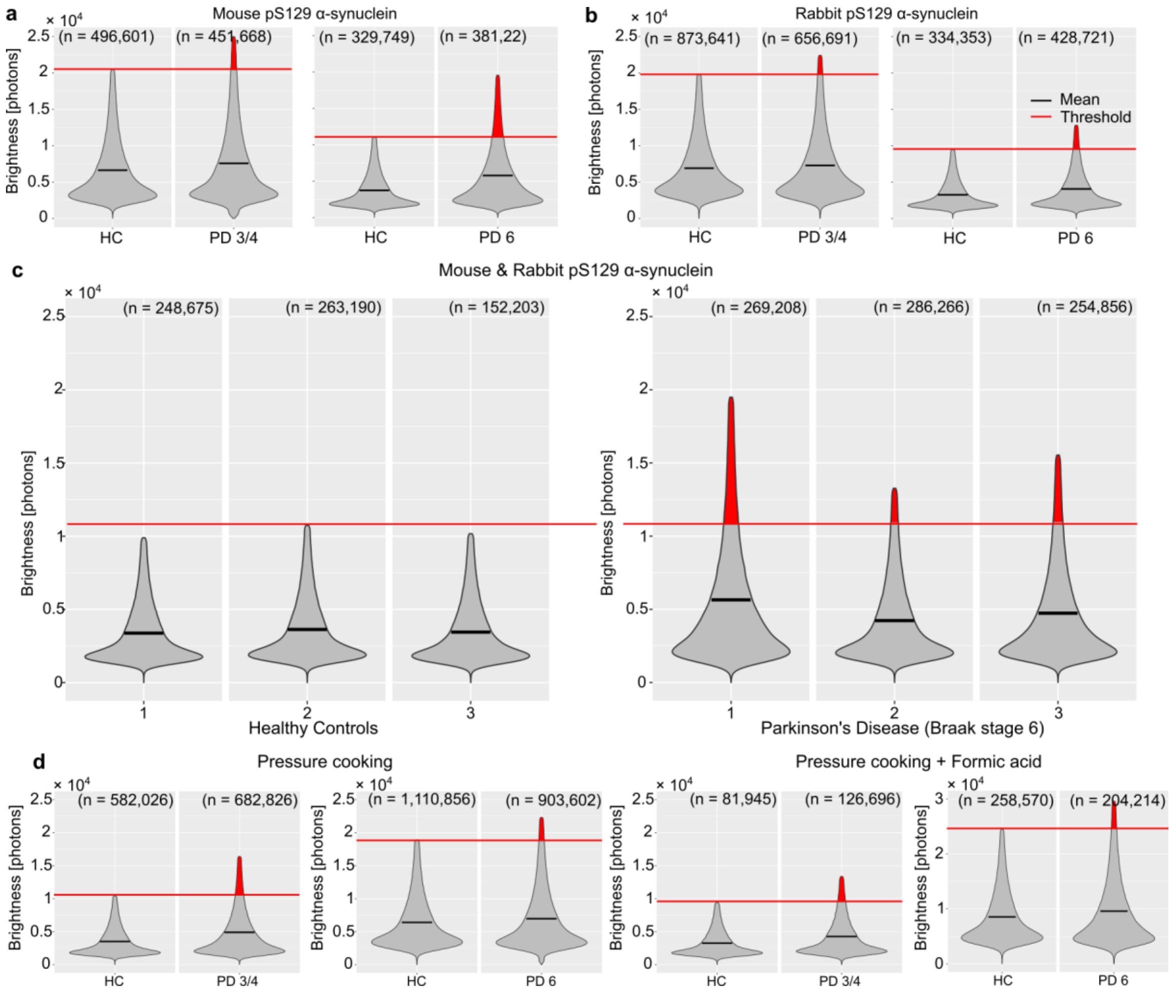

Supplementary Fig. S15: Nanoscale-assembly brightnesses in PD and HC samples are different across a range of conditions. **a.b.** Violin plots of the brightnesses detected in $n = 3$ HC, $n = 3$ PD Braak stage 3/4 and n=3 PD Braak stage 5/6 (**a**) Disease-specific (bright) species can be detected using the mouse (AB_2819037) and rabbit pS129 α-synuclein (AB_2270761) antibodies, respectively, across both mid- and -late-stage disease. (**b**) Braak stage 6 patients using formalin fixation and paraffin embedding (FFPE). **c.** Violin plots of the intensity (photons) of aggregates detected in $n = 3$ HC and $n = 3$ PD (Braak stage 6) patients detected using both α-synuclein antibodies (AB_2819037 & AB_2207061). Disease-specific species are consistent across all individuals. Samples were either treated with a pressure cooker only or pre-treated with formic acid and pressure cooked. Disease-specific aggregates are observed across pre-treatment conditions. All data plots are truncated at 1.5 x IQR. Distributions were compared by two-sample K-S tests, *** P<0.001.

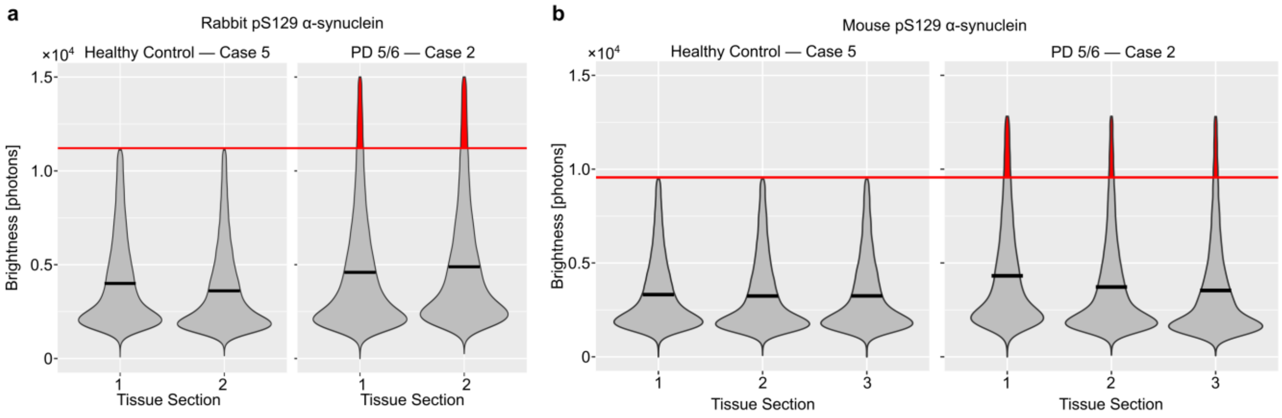

Supplementary Fig. S16: Reproducibility of ASA-PD signal across adjacent tissue sections from the same case. (a) Violin plots of ASA-PD intensity show variability in nano-assembly brightness between adjacent sections from the same individual (HC: left; PD: right), while the disease-associated subpopulation remains consistently represented. **(b)** A comparable repeatable pattern is observed when using antibodies raised in different host species, applied to adjacent sections from the same case (HC: left; PD: right). In all instances, tissue demonstrates a robust and reproducible signal that systemically is higher disease vs control.

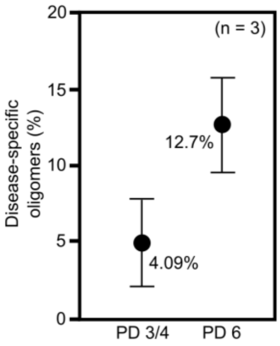

Supplementary Fig. 17: Disease-specific species increase with Braak staging. The percentage of disease-specific species increases by 8.61% in late-stage Braak stage 5/6 PD cases compared to mid-stage Braak 3/4 cases indicating that disease-specific species increase as disease progresses.

3.4: Properties of ASA-PD in comparison to orthogonal aggregate detection methods

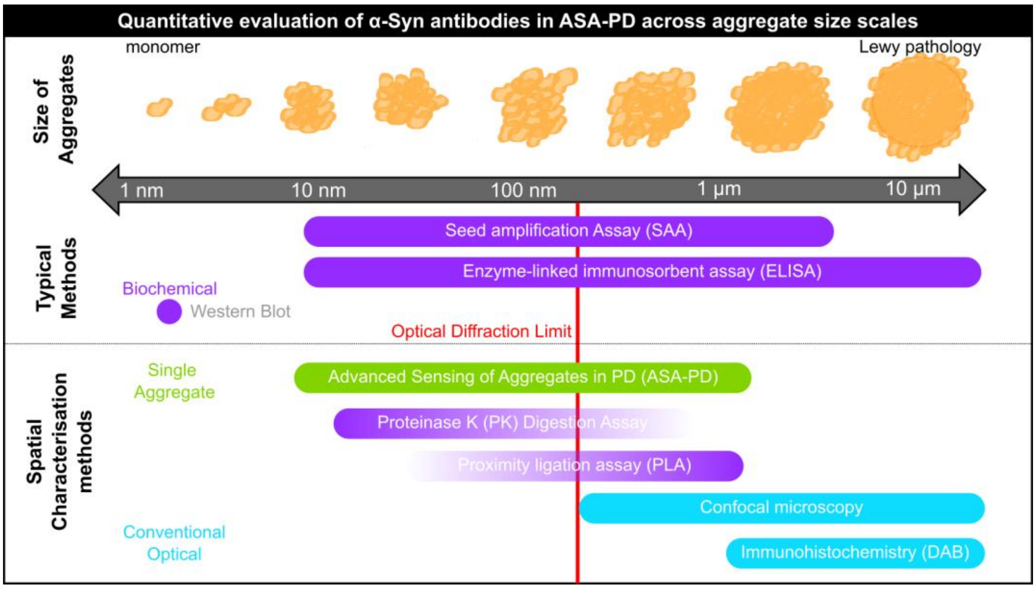

Supplementary Figure S18: Sensitivity of methods to characterize the entire distributions of α-synuclein aggregation. Monomeric α-synuclein initially assembles into small objects before advancing to Lewy-related pathology. Various orthogonal methods characterize these stages, including biochemical techniques (blue), microscopy-based approaches (blue), and our ASA-PD method (green), each suited to different spatial scales, as shown in the lower panel. S

When selecting an analytical method to examine α-synuclein, it is important to not only consider the spatial scale for which each technique is optimised across the continuum of aggregate sizes. We have summarized *typical* size values in Supplementary Figure S18. The methods include:

- **Proximity ligation assay (PLA)**, which identifies protein-protein interactions by amplifying signals from proximity-labelled antibodies, typically resolving protein complexes at the nanoscale (~10–100 nm). The amplification probability is proportional to aggregate size; therefore, larger aggregates can be disproportionately amplified over the smallest molecular assemblies.
- **Enzyme-linked immunosorbent assay (ELISA)**, which quantifies proteins or antigens using antibody-based detection. They are best suited for bulk measurements and typically optimized for the 10nm –1μm scale.
- **Seed application assay (SAA)**, which assesses the ability of misfolded proteins to induce aggregation in a biological sample, typically detecting fibrillar species in the microscale due to the amplification step.
- **Western blotting**, which detects specific proteins through gel electrophoresis, membrane transfer, and antibody staining, working effectively at the monomer to oligomer scale.
- **Proteinase K digestion**, which degrades proteins to evaluate their resistance to proteolysis, commonly used in prion and amyloid research, and is informative for aggregates spanning small assemblies to large fibrils (10 nm to several microns). Importantly, the relative signal change is most informative at the smallest scale, allowing differentiation of structural order within aggregates.
- **Confocal microscopy**, which provides high-resolution imaging of fluorescently labelled samples, optimized for structures above the diffraction limit (~200 nm–10 μm).
- **Immunohistochemical DAB staining**, which visualises specific proteins in tissue sections using enzyme-linked antibodies that generate a brown precipitate, best suited for detecting deposits and inclusions at the microscale (~1–100 μm).

While **ASA-PD** has been optimised to specifically target low-contrast, smaller aggregates, single proteins remain undetectable due to the autofluorescence background floor. Based on single-molecule quantification experiments, we estimate that the smallest detectable assemblies in the current implementation are likely larger than 5-mers, though this could be improved with better background-reduction methods.

We selected the PLA and SAA methods to provide orthogonal validation methods to ASA-PD for the detection of aggregates.

Proximity Ligation Assay of α-synuclein reveals enriched population of aggregated α-synuclein in PD

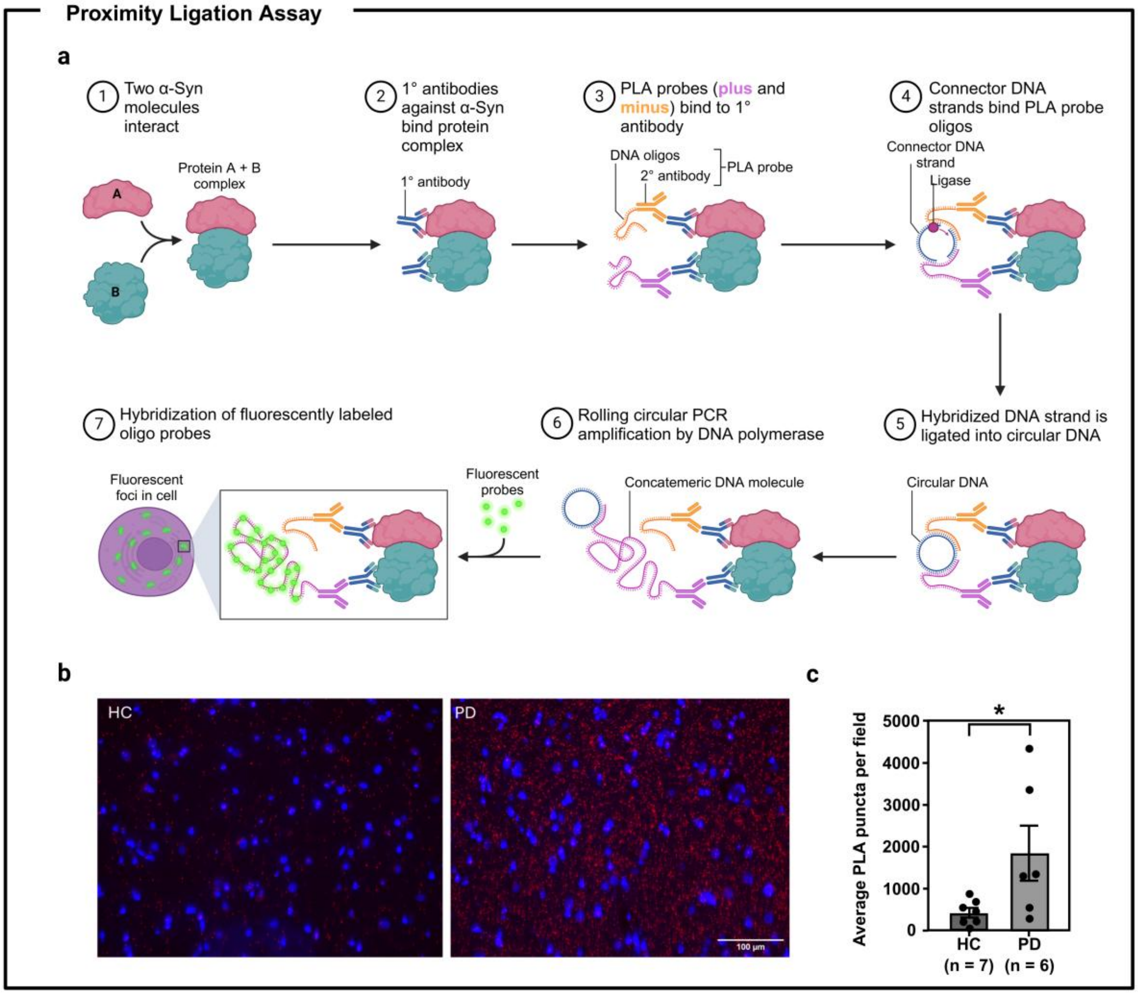

Supplementary Figure S19: Increased PLA signal in PD cingulate cortex compared to HC. a) Schematic illustrating the method that is used for detecting proximity ligation signals. Two antibodies have to be bound close enough together to initiate a fluorescent signal. b) Red dots are positive diffuse PLA signals. Blue indicates DAPI as a nuclei stain. Scale bar represents 100µm. c) Average number of puncta per field of view for each case measured. Mann-Whitney U test detects significant difference at level p=0.0350, indicated by *. Created in BioRender. Evans, J. (2025) https://BioRender.com/2ssl0qt.

Seed Amplification reveals the presence of seed competent aggregates in PD tissue

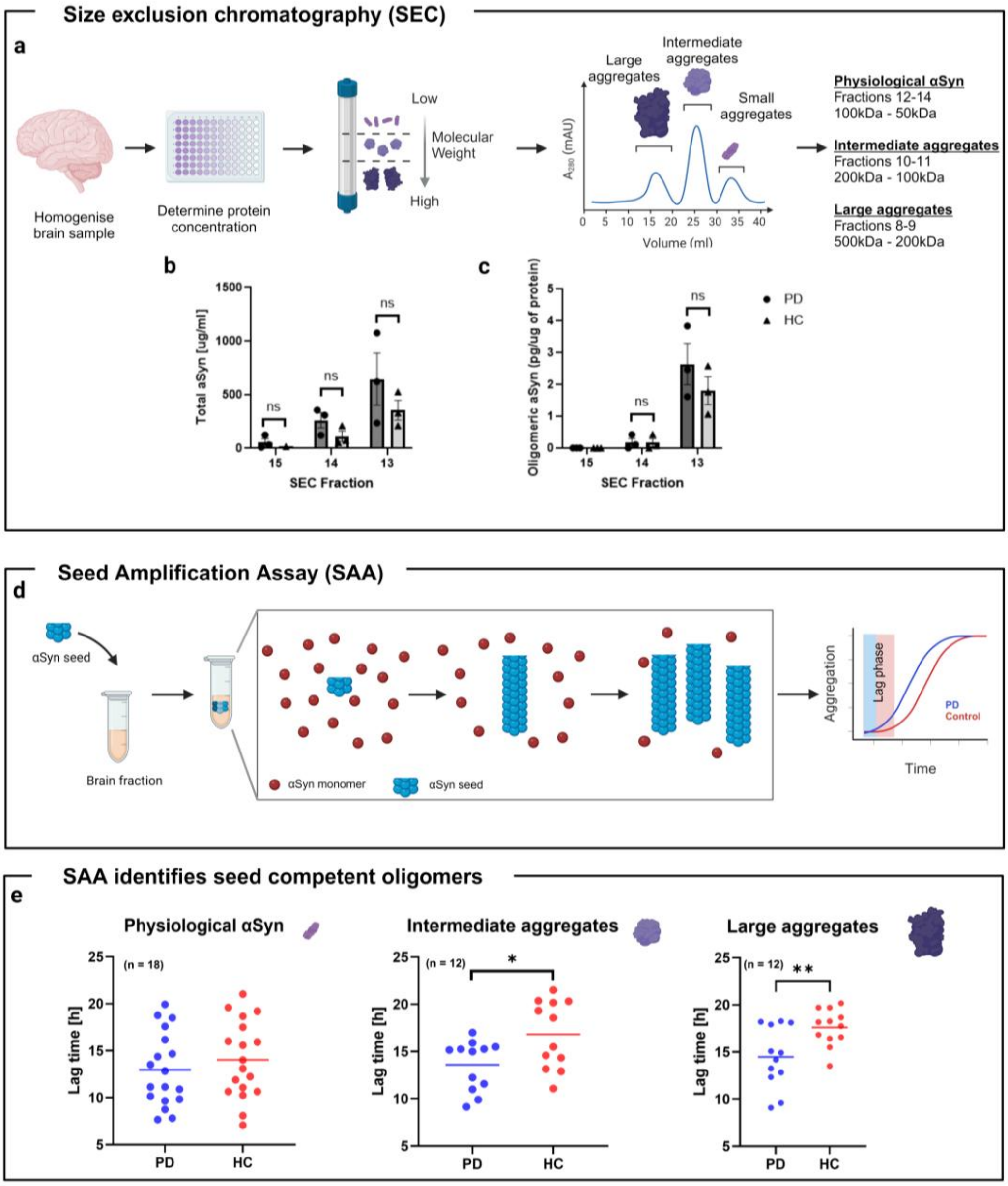

Supplementary Figure S20: Seed competent assemblies present in PD brain tissue. a) Schematic illustrating how size exclusion chromatography (SEC) was used to separate out brain homogenate from cingulate cortex of PD and control cases according to molecular weight. Briefly, brain tissue was homogenised, protein concentration determined by BCA protein assay and equal quantities of protein loaded into size exclusion column. Chromatograms indicate which molecular weights come off column over time. Three groups of fractions were taken forward for further analysis: Large aggregates (fractions 8-9), Intermediate aggregates (fractions 10-11) and physiological α-synuclein (fractions 12-14). b) Concentration of total αSyn as indicated by ELISA for fractions 13-15 in PD and HC groups. Two-way ANOVA revealed no significant difference between disease groups). Error bars indicate standard error of the mean. c) Oligomeric αSyn levels in SEC fractions 13-15 as indicated by sandwich ELISA in PD and HC groups. Two-way ANOVA determined no significant difference between disease groups. Error bars indicate standard error of the mean. d) Schematic illustrating how seed amplification assay for α-synuclein was performed on fractions obtained from SEC. Briefly each SEC fraction was incubated with a recombinant α-synuclein seed, agitated alongside fluorescent ThT and the level of aggregation observed over time as a measure of fluorescence. The shorter the lag phase the quicker aggregation of α-synuclein has occurred. e) Lag times for three molecular weight groups in PD and controls. PD cases had significantly smaller lag times in the large aggregate group (p=0.0091) and intermediate aggregate group (p=0.0203) when compared to controls. No significance was observed in the small aggregate group (0.4337). Groups were not compared to each other due to differing alpha-synuclein concentrations. Significance is indicated with **. Created in BioRender.com.

Sensitivity of aggregates to Proteinase K digestion

We used Proteinase K digestion to determine if the fluorescent puncta in our samples were enzyme-resistant as has been reported previously for assemblies[19] (Supplementary Figure S21). In tissue samples treated with Proteinase K 50µg/mL and then labelled, we observe a significant decrease in the number of aggregates; however, some aggregates remain detectable even after two minutes indicating a resistant α-synuclein subpopulation and shows ASA-PD detects both Proteinase K-sensitive and Proteinase K-resistant species. Specifically, we observe a reduction in the disease-associated bright aggregates within 10 seconds of treatment alongside a decrease in physiological dimmer species.

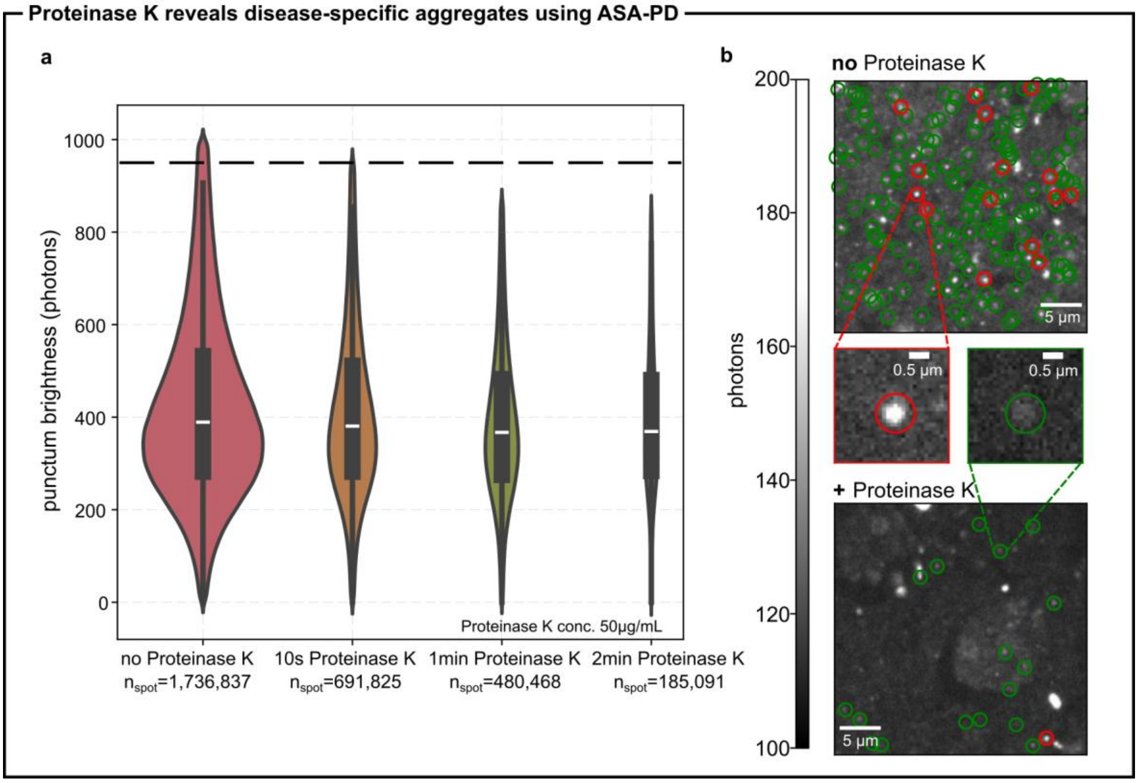

Supplementary Figure S21: Proteinase K Assay reveals degradation resistance subpopulations **a**. The quantification of imaged PD tissue with various durations of 50 ug/mL Proteinase K treatment. After treatment, samples were washed, labelled, and characterised as normal. **b**. Example images pre-Proteinase K treatment and post. Pre-treatment shows brighter (red) and dimmer (green) assemblies, at higher density than post Proteinase K treatment. Zoom-in shows a representative image of the existence of PK resistant puncta consistent with the brighter assemblies found in PD tissue.

4. Object density with segmented cells

The densities of the bright assembly subpopulation found in PD tissue, was measured in samples co-stained with various cell markers are characterized in Supplementary Table 6. In brief, the number of nanoscale bright objects in each field of view that overlapped with each segmented cell-marker images were normalized by the measured density in the field of view. Specifically, the *weighted density* (row 9) was calculated as the ratio of the *density of bright species per field of view* (row 2) to the *density of cell-specific bright species per segmented area* (row 8). This ratio accounts for sampling variations between different fields of view, where a value of 1 indicates no difference between the inside-cell and whole field-of-view density. The corresponding t-value is reported in row 10.

5. Supplementary Tables

Supplementary Table 1. Commerical antibodies tested for ASA-PD

Antibody	RRID/CAS	Catalogue code	Epitope
**Rabbit polyclonal pS129** alpha-**synuclein**	AB_227076	AB59264	pS129
Mouse monoclonal pS129 alpha-synuclein	AB_2819037	AB184674	pS129
Rabbit monoclonal pS129 alpha-synuclein [EP1536Y]	AB_869973	AB51253	pS129
Rabbit monoclonal alpha-synuclein aggregate (MJFR14)	N/A	AB209583	“Aggregated α-synuclein”
Mouse monoclonal alpha-synuclein (syn-211)	AB_628318	sc-12767	AA 121-125
Mouse monoclonal alpha-synuclein (LB509)	AB_2832854	Ab27766	AA 1150121
Mouse monoclonal alpha synuclein 34-45	AB_2650703	849101	AA 34-45
Mouse monoclonal alpha synuclein 80-96	N/A	2650687	AA 80 96
Mouse monoclonal alpha synuclein 103-108	AB_2734561	807804	AA 103-108
Rabbit monoclonal alpha-synuclein (MJFR1)	AB_2537217	AB138501	AA 118-123

[1]Several antibodies against different epitopes on α-synuclein were qualitatively assessed for their applicability in ASA-PD. Suitability is here defined as: (1) the density of labelled protein gives rise to spatially isolated diffraction-limited fluorescent puncta in the image, and (2) the antibody does not affect the magnitude of the autofluorescence background, this is confirmed with a secondary-antibody only control.

Supplementary Table 2. Summary of case demographics for human post-mortem brain tissue ASA-PD staining

	Healthy Control cases	PD, Braak stage 3/4	PD Braak stage 6
**Number of cases**	6	3	3 (+1 neg control only)
**Brain bank**	3 QSBB, 3 Imperial	Imperial	QSBB
**Age at death**	87 (71-101)	80 (73-85)	75 (73-84)
**Sex**	M	M	M
**Post-mortem interval [hours][1]**	45 (18-98)	21 (16-24)	68 (38-89)

[1]Age at death and PMI are given as a mean with range in brackets. PMI - Post-mortem Interval. Negative control is a case that was only used for technical control rather than the main study.

Supplementary Table 3 Case Demographics

Case	*Brain Bank[1]*	*Pathological diagnosis[1]*	*Age*	*Sex*	*Onset*	*Duration*	*PMI (hours)*	*Confounding pathology*	*Braak score (α-syn)*	*Braak and Braak score (tau)*	*Thal score (amyloid β)*
**1***	QSBB	PD	81	M	50	31	73	mild small vessel disease, microscopic cerebellar infarction, leptomeningeal vessel atheroma	6	2	2
**2***	QSBB	PD	84	M	61	23	71	mild CAA, hyaline arteriosclerosis, ARTAG	6	1	1
**3***	QSBB	PD	63	M	42	21	38	mild small vessel disease	6	1	0
**4***	QSBB	PD	73	M	62	11	89	ARTAG, microinfarcts, CAA, hyaline arteriosclerosis, atherosclerosis	6	1	3
**5***	QSBB	Control	87	M	-	-	40	moderate small vessel disease	0	1	0
**6***	QSBB	Control	101	M	-	-	60	ARTAG, CAA, hyaline arteriosclerosis, atherosclerosis	0	1	0
**7***	QSBB	Control	88	M	-	-	98	Pathological ageing, CAA	0	2	2
**8***	Imperial	PD	73	M			24		3	1	3
**9***	Imperial	PD	81	M			22		4	2	
**10***	Imperial	PD	85	M	70	15	16		4	2	
**11***	Imperial	Control	71	M	-	-	29	Pathological ageing	0	-	-
**12***	Imperial	Control	91	M	-	-	18	PART, ARTAG	0	-	-
**13***	Imperial	Control	85	M	-	-	23	Pathological ageing	0	-	3
**14#**	Imperial	Control	83	M	-	-	21		0	-	-
**15#**	Imperial	Control	76	F	-	-	22		0		
**16#**	Imperial	Control	84	M	-	-	9		0		
**17#**	Imperial	Control	91	F	-	-	22		0		
**18#**	Imperial	Control	92	F	-	-	24		0		

**19#**	Imperial	Control	90	F	-	-	22	0
**20#**	Imperial	Control	82	M	-	-	20	0
**21#**	Imperial	PD	77	M	57	20	23	6
**22#**	Imperial	PD	86	F	77	9	23	6
**23#**	Imperial	PD	78	M	65	13	19	5
**24#**	Imperial	PD	79	F	72	7	15	5
**25#**	Imperial	PD	73	M	49	24	2	6
**26#**	Imperial	PD	88	F	78	10	14	5

[1]PD, Parkinson's disease; QSBB, Queen Square Brain Bank; PMI, Post-mortem Interval; * cases used for main study; # cases used for PLA

Supplementary Table 4: Staining Plan Table

Sample	Case	Disease	Region	Pretreatment	Primary antibodies	Secondary antibodies
1.01-1.03	8,9,10	PD	ACG	PC+FA	mou LB509 and rab p-syn	mou 488 and rab 568
1.04-1.06	11,12,13	Control	ACG	PC+FA	mou LB509 and rab p-syn	mou 488 and rab 568
1.07-1.09	8,9,10	PD	ACG	PC	mou LB509 and rab p-syn	mou 488 and rab 568
1.10-1.12	11,12,13	Control	ACG	PC	mou LB509 and rab p-syn	mou 488 and rab 568
1.13	4	PD	ACG	PC	none	mou 488 and rab 568
1.14	4	PD	ACG	PC+FA	none	mou 488 and rab 568
1.15	4	PD	ACG	PC	none	rab 568
1.16	4	PD	ACG	PC+FA	none	rab 568
2.01-2.03	8,9,10	PD	ACG	PC	rab OLIG2 and mou p-syn	rab 488 and mou 568
2.04-2.03	11,12,13	Control	ACG	PC	rab OLIG2 and mou p-syn	rab 488 and mou 568
2.07-2.09	8,9,10	PD	ACG	PC	mou NF and rab p-syn	mou 488 and rab 568
2.10-2.12	11,12,13	Control	ACG	PC	mou NF and rab p-syn	mou 488 and rab 568
2.13	4	PD	ACG	PC	none	mou 488
2.14	4	PD	ACG	PC	none	rab 488
2.15	4	PD	ACG	PC	none	mou 568
2.16	4	PD	ACG	PC	none	rab 568
3.01-3.03	8,9,10	PD	ACG	PC	mou GFAP and rab p-syn	mou 488 and rab 568
3.04-3.06	11,12,13	Control	ACG	PC	mou GFAP and rab p-syn	mou 488 and rab 568
3.07-3.09	8,9,10	PD	ACG	PC	rab P2RY12 and mou p-syn	rab 488 and mou 568
3.10-3.12	11,12,13	Control	ACG	PC	rab P2RY12 and mou p-syn	rab 488 and mou 568
3.13	4	PD	ACG	PC	NONE	mou 488
3.14	4	PD	ACG	PC	NONE	rab 568
3.15	4	PD	ACG	PC	NONE	rab 488
3.16	4	PD	ACG	PC	NONE	mou 568
7.01-7.03	1,2,3	PD	ACG	PC+FA	mou LB509 and rab p-syn	mou 488 and rab 568
7.04-7.06	5,6,7	Control	ACG	PC+FA	mou LB509 and rab p-syn	mou 488 and rab 568
7.07-7.09	1,2,3	PD	ACG	PC	mou LB509 and rab p-syn	mou 488 and rab 568
7.10-7.12	5,6,7	Control	ACG	PC	mou LB509 and rab p-syn	mou 488 and rab 568
7.13-7.15	1,2,3	PD	ACG	PC	mou NF and rab p-syn	mou 488 and rab 568
7.16-7.18	5,6,7	Control	ACG	PC	mou NF and rab p-syn	mou 488 and rab 568
7.19	4	PD	ACG	PC	none	mou 488 and rab 568
7.20	4	PD	ACG	PC+FA	none	mou 488 and rab 568
7.21	4	PD	ACG	PC	none	rab 568
7.22	4	PD	ACG	PC+FA	none	rab 568
8.01-8.03	1,2,3	PD	ACG	PC	rab P2RY12 and mou p-syn	rab 488 and mou 568
8.04-8.06	5,6,7	Control	ACG	PC	rab P2RY12 and mou p-syn	rab 488 and mou 568
8.07-8.09	1,2,3	PD	ACG	PC	rab OLIG2 and mou p-syn	rab 488 and mou 568
8.10-8.12	5,6,7	Control	ACG	PC	rab OLIG2 and mou p-syn	rab 488 and mou 568
8.13-8.15	1,2,3	PD	ACG	PC	mou GFAP and rab p-syn	mou 488 and rab 568
8.16-8.18	5,6,7	Control	ACG	PC	mou GFAP and rab p-syn	mou 488 and rab 568
8.19	4	PD	ACG	PC	none	mou 488
8.20	4	PD	P CTX	PC	none	rab 488

8.21	4	PD	P CTX	PC	none	mou 568
8.22	4	PD	P CTX	PC	none	rab 568

[1]Case numbers refer to those in Table 1. ACG – anterior cingulate gyrus; P CTX – parietal cortex; PC – pressure cook in citrate buffer; FA – formic acid; SB – Sudan Black B; all samples embedded in formalin fixed paraffin.

Supplementary Table 5 Reagents and tools.

Item	Catalog ID	Stock concentration	Dilution factor	Concentration used (mg/ml)	RRID/CAS
rabbit polyclonal to α-synuclein (phospho S129)	AB59264	1 mg/ml	1:200	0.005	AB_2270761
mouse monoclonal (psyn/81A) to α-synuclein (phospho S129)	AB184674	1 mg/ml	1:500	0.002	AB_2819037
LB509	807709	0.5 mg/ml	1:100	0.005	AB_2832854
GFAP	AB190288	1 mg/ml	1:1000	0.001	AB_2747779
OLIG2	AB9610	0.55 mg/ml	1:100	0.0055	AB_570666
P2RY12	HPA014518	0.2 mg/ml	1:100	0.00200	AB_2669027
Neurofilament [RT-97]	ab178589	0.2 mg/ml	1:200	0.00100	AB_2941917
TARDBP [2E2-D3]	H00023435-M01	10 µg/ml	1:800	0.000013	AB_425904
Anti- Tau (4-repeat isoform RD4), Clone 1E1/A6	05-804	-	1:2000/1:3000	--	AB_310014
Phospho-Tau (Ser202, Thr205) Monoclonal Antibody (AT8)	MN1020	0.2 mg/ml	1:600	0.00033	AB_223647
Ubiquitin	Z0458	-	-	-	AB_2315524
P62	610833	250 µg/ml	-	-	AB_398152
Antibody, Alexa Fluor 568 goat anti-mouse – A11031	A11031	2 mg/ml	1:200	0.01	AB_144696
Antibody, Alexa Fluor 568 goat anti-rabbit – A11011	A11011	2 mg/ml	1:200	0.01	AB_143157
Antibody, Alexa Fluor 488 goat anti- rabbit – A11008	A11008	2 mg/ml	1:200	0.01	AB_143165
Antibody, Alexa Fluor 488 goat anti-mouse – A11001	A11001	2 mg/ml	1:200	0.01	AB_2534069
Rabbit monoclonal alpha-synuclein (MJFR1)					
TetraSpeck beads	T7279	-	-	-	-
Methylated Spirit	IMS005	-	-	-	64-17-5
Xylene	XYL005	-	-	-	1330-20-7
Methanol	8222835000	-	-	-	67-56-1
Hydrogen Peroxide	23615.261	-	-	-	7722-84-1
PBS tablets	18912014	-	-	-	7647-14-5
VECTASHIELD+ PLUS Antifade Mounting Medium	H-1900-10	-	-	-	AB_2336789
Tri-sodium Citrate (for antigen retrieval)	27830.294	-	-	-	6132-04-3
Citric Acid (for antigen retrieval)	C/6200/53	-	-	-	5949-29-1
Sudan Black (Concentrate)	199664-25G	-	-	-	4197-25-5
Superfrost Plus microscope slide, Fisherscientific	4951PLUS602811				-
Coverslip 22x50 mm #1, VWR	631-0137				-
24 x 50 mm, #1, VWR	48404-453				-
CoverGrip™ sealant, Biotium,	23005				-
Ar plasma cleaner, Harrick Plasma	PDC-002				PDC-002-HP (230V)
CultureWell™ Reusable Gasket, 6mm diameter, Grace Bio-Labs	103280				
Poly-L-Lysine, Sigma-Aldrich	P4707				-
PBS (pH 7.4, 1x Gibco, Thermo Fisher Scientific	10010023				-
488 Laser, Toptica	488 iBeam-SMART				60825-1:2014

561 Laser, Oxxius	561 LaserBoxx, DPSS		LAS-05989
100x, NA 1.49 oil-immersion Objective, Nikon	Nikon CFI Apo TIRF 100XC Oil		NIKON-MRD01991
Multiband dichroic filter, Semrock	Di01-R405/488/561/635		FL-007046
Emission bandpass filter, Semrock	520/44-25		FL-004678
Excitation bandpass filter, Semrock	BLP01-488R		FL-008552
Emission longpass filter, Semrock	LP02-568RS-25		-
Excitation bandpass filter, Semrock	FF01-587/35-25		FL-004312
EMCCD, Photometrics	Evolve 512 Delta		-
ImageJ	1.53q		SCR_003070
Micromanager	2		SCR_016865
MATLAB	R2023b		SCR_001622
GDSC SMLM	1		SCR_022717
Origin	10.0		SCR_014212
BioRender	2023		SCR_018361
Python	3.5		SCR_008394

Supplementary Table 6 Oligomer-cell density.

Costain	Neurons (neurofilament)	Microglia (p2ry12)	Astrocytes (gfap)
Number of bright oligomers per field of view	8.4±0.9	30±2	10.7±0.9
Density of bright oligomers per field of view (μm^{-2})	0.0028±0.0003	0.0099±0.0008	0.0036±0.0003
Number of cell specific bright oligomer per field of view	0.74±0.14	1.20±0.20	0.78±0.13
Cell specific bright oligomer per field of view (%)	8.80±1.52	4.03±0.74	7.36±1.27
Cell area per field of view (μm^2)	181.68±12.31	101.96±6.56	196.75±12.96
Cell area per field of view (%)	6.05±0.41	3.40±0.22	6.56±0.43
Whole Cell area (mm^2)	0.198	0.123	0.260
Density of cell-specific bright oligomers per segmented area	0.00408±0.0009	0.0118±0.0023	0.00390±0.0009
Weighted density	1.453±0.35	1.187±0.25	1.123±0.27
Statistical difference from a random distribution t-value	1.30	0.74	0.46

6. Supplementary References

92. Peng, C. *et al.* Cellular milieu imparts distinct pathological α-synuclein strains in α-synucleinopathies. *Nature* **557**, 558–563 (2018).
93. Moerner, W. E. & Fromm, D. P. Methods of single-molecule fluorescence spectroscopy and microscopy. *Review of Scientific Instruments* **74**, 3597–3619 (2003).
94. Otsu, N. A Threshold Selection Method from Gray-Level Histograms. *IEEE Trans. Syst., Man, Cybern.* **9**, 62–66 (1979).
95. Haralick, R. M., Sternberg, S. R. & Zhuang, X. Image Analysis Using Mathematical Morphology. *IEEE Trans. Pattern Anal. Mach. Intell.* **PAMI-9**, 532–550 (1987).
