# Supplementary material for: Large-scale visualization of α-synuclein oligomers in Parkinson’s disease brain tissue
Source: Nat Biomed Eng. 2025 Oct 1;10(6):1222–34. doi: 10.1038/s41551-025-01496-4 (PMC13278957; doi:10.1038/s41551-025-01496-4)
Supplement: Supplementary file 2 — Reporting Summary [file 41551_2025_1496_MOESM2_ESM.pdf]

nature portfolio

Corresponding author(s): Lucien E. Weiss, Sonia Gandhi, Steven F. Lee

Last updated by author(s): Jul 16, 2024

Reporting Summary

Nature Portfolio wishes to improve the reproducibility of the work that we publish. This form provides structure for consistency and transparency in reporting. For further information on Nature Portfolio policies, see our Editorial Policies and the Editorial Policy Checklist.

Statistics

For all statistical analyses, confirm that the following items are present in the figure legend, table legend, main text, or Methods section.

n/a	Confirmed	
☐	☒	The exact sample size (*n*) for each experimental group/condition, given as a discrete number and unit of measurement
☐	☒	A statement on whether measurements were taken from distinct samples or whether the same sample was measured repeatedly
☐	☒	The statistical test(s) used AND whether they are one- or two-sided<br>*Only common tests should be described solely by name; describe more complex techniques in the Methods section.*
☐	☒	A description of all covariates tested
☐	☒	A description of any assumptions or corrections, such as tests of normality and adjustment for multiple comparisons
☐	☒	A full description of the statistical parameters including central tendency (e.g. means) or other basic estimates (e.g. regression coefficient) AND variation (e.g. standard deviation) or associated estimates of uncertainty (e.g. confidence intervals)
☐	☒	For null hypothesis testing, the test statistic (e.g. *F*, *t*, *r*) with confidence intervals, effect sizes, degrees of freedom and *P* value noted<br>*Give P values as exact values whenever suitable.*
☒	☐	For Bayesian analysis, information on the choice of priors and Markov chain Monte Carlo settings
☒	☐	For hierarchical and complex designs, identification of the appropriate level for tests and full reporting of outcomes
☒	☐	Estimates of effect sizes (e.g. Cohen's *d*, Pearson's *r*), indicating how they were calculated

Our web collection on statistics for biologists contains articles on many of the points above.

Software and code

Policy information about availability of computer code

Data collection	We use micromanager (version 2.0.0) for image acquisition.
Data analysis	We use MATLAB (version 2022a) for image processing and image analysis, and Inkscape (version 1.3.2) for figure plotting

For manuscripts utilizing custom algorithms or software that are central to the research but not yet described in published literature, software must be made available to editors and reviewers. We strongly encourage code deposition in a community repository (e.g. GitHub). See the Nature Portfolio guidelines for submitting code & software for further information.

Data

Policy information about availability of data

All manuscripts must include a data availability statement. This statement should provide the following information, where applicable:

- Accession codes, unique identifiers, or web links for publicly available datasets
- A description of any restrictions on data availability
- For clinical datasets or third party data, please ensure that the statement adheres to our policy

All unprocessed data, processed data (binary masks, density and intensity information), methods, and detailed protocols are available online, which can be found in Materials and Methods section in the paper.

Research involving human participants, their data, or biological material

Policy information about studies with human participants or human data. See also policy information about sex, gender (identity/presentation), and sexual orientation and race, ethnicity and racism.

Reporting on sex and gender	As this study was developing methods for visualising small oligomeric structures, we designed our study to focus on analysis of technical variables rather than confounding biological variables where possible. For this reason only human post-mortem brain samples of Male sex were used for the study. Further studies that use this method in more detail will endeavour to use both Female and Male sexes to control for any biological differences between the two. The sex of the human post-mortem samples were obtained from the pathology reports the brain banks issued when the tissue samples were received (Queen Square Brain Bank for Neurological disorders, University College London and Multiple Sclerosis and Parkinson's Brain Bank, Imperial College London). All human post-mortem brain tissue provided has been donated with informed consent.
Reporting on race, ethnicity, or other socially relevant groupings	Data on race, ethnicity and other socially relevant groupings are not routinely collected by each of the brain banks the human tissue were obtained from. We therefore did not include these variables or describe these features within our study design or demographic table.
Population characteristics	We have information about the age of death of all human post-mortem brain samples and the clinical and pathological diagnoses these cases have based on the reports received from both brain banks. Our study design matched as close as possible the age of the study groups being compared. The pathological diagnosis of each case were used to determine whether the case was classed as having Parkinson's disease or a healthy control. The Braak staging criteria that stages the level of alpha-synuclein pathology in the post-mortem brain was used to identify the severity of disease present within each brain and cases were separated into early-mid stage Parkinson's disease (Braak stage 3/4) and late stage Parkinson's disease (Braak stage 6). Other pathological scores for co-pathologies relevant to Alzheimer's disease or dementia (Braak and Braak staging and Thal staging) were also obtained and used to select which cases were used within the study. The cases selected for the study had the lowest staging of co-pathologies present.
Recruitment	Human post-mortem brain tissue is donated to the relevant brain bank. Each brain bank has their own recruitment practices for donors but this is not communicated when requesting the tissue.
Ethics oversight	Ethical approval for the study was covered under the ethics granted for research on the brain tissue from the Local Research Ethics committee of the National Hospital for Neurology and Neurosurgery (23/LO/0044).

Note that full information on the approval of the study protocol must also be provided in the manuscript.

Field-specific reporting

Please select the one below that is the best fit for your research. If you are not sure, read the appropriate sections before making your selection.

☒ Life sciences ☐ Behavioural & social sciences ☐ Ecological, evolutionary & environmental sciences

For a reference copy of the document with all sections, see nature.com/documents/nr-reporting-summary-flat.pdf

Life sciences study design

All studies must disclose on these points even when the disclosure is negative.

Sample size	Sample groups of 3 were used for each comparison group to control for any technical variability allowing anomalies within the data to be determined. We focused the study design on comparison of technical sample groups as we were developing our method. Any differences identified were used to refine the method. Biological variation between groups are being used as preliminary data and will be investigated in more detail now the method is more established.
Data exclusions	A quality control step was performed on automatically acquired images to reject out-of-focus data. No samples were excluded.
Replication	The number of samples and patients are reported in table.
Randomization	From the samples we could have access to from each brain bank, we selected cases to control for confounding biological variables as much as possible. Cases were not chosen randomly but were chosen to be age and sex matched between study groups. Healthy controls and late stage Parkinson's disease brains were seperated based upon pathological staging scores. Randomisation of the images taken for each sample are described in more detail within the methods section of the manuscript.
Blinding	Sample were automatically analysed with the same processing pipeline using the same input parameters. Samples were not blinded.

Reporting for specific materials, systems and methods

We require information from authors about some types of materials, experimental systems and methods used in many studies. Here, indicate whether each material, system or method listed is relevant to your study. If you are not sure if a list item applies to your research, read the appropriate section before selecting a response.

Materials & experimental systems

n/a	Involved in the study
☐	☒ Antibodies
☒	☐ Eukaryotic cell lines
☒	☐ Palaeontology and archaeology
☐	☒ Animals and other organisms
☒	☐ Clinical data
☒	☐ Dual use research of concern
☒	☐ Plants

Methods

n/a	Involved in the study
☒	☐ ChIP-seq
☒	☐ Flow cytometry
☒	☐ MRI-based neuroimaging

Antibodies

Antibodies used

All antibody details are listed in detail in Supplementary Table 4.

Validation

AB_2270761 - Abcam statement. We have tested this species and application combination and it works. It is covered by our product promise. doi: 10.1186/s40478-014-0164-0

AB_2819037- Abcam statement. We have tested this species and application combination and it works. It is covered by our product promise. doi: 10.1038/srep42984

AB_2832854 - Each lot of this antibody is quality control tested by formalin-fixed paraffin-embedded immunohistochemical staining. This antibody reacts with human, but does not react with rodent a-synuclein. The antibody recognizes amino acids 115-122 of human a-synuclein. doi: 10.1073/pnas.1309143110

AB_2747779 - Abcam statement. We have tested this species and application combination and it works. It is covered by our product promise.

AB_570666 - Merck. Evaluated by immunohistochemistry on glioblastoma. Immunohistochemistry (Paraffin): Representative lot data. Anti-Olig2 (AB9610) staining pattern morphology in glioblastoma. Tissue was pretreated with TE Buffer, pH 9.0. Polyclonal antibody was diluted to 1:500, using IHC-Select® Detection with HRP-DAB.

AB_2669027 - Merck Enhanced validation - orthogonal RNAseq. All Prestige Antibodies Powered by Atlas Antibodies are developed and validated by the Human Protein Atlas (HPA) project and as a result, are supported by the most extensive characterization in the industry.

AB_425904 - ThermoFisher Scientific Antibody Performance Guarantee. Tested in multiple applications. Immunoperoxidase of monoclonal antibody to TARDBP on formalin-fixed paraffin-embedded human leiomyosarcoma tissue Antibody concentration is 3 µg/mL

AB_310014 - Merck. Immunohistochemistry: This antibody has been reported by an independent laboratory to detect Tau (4-repeat isoform RD4) in autoclaved paraffin brain sections. (De Silva, R., 2003;Togo, T., 2002)

AB_223647 - ThermoFisher Scientific Advanced Verification. This Antibody was verified by Cell treatment to ensure that the antibody binds to the antigen stated. doi: 10.1038/s41596-023-00861-4

AB_2315524 - Dako. doi: 10.1016/j.ajpath.2017.10.021

AB_398152 - doi: 10.1093/jnen/nlad051

AB_144696, AB_143157, AB_143165, AB_2534069 - ThermoFisher Scientific Antibody Performance Guarantee. To minimize cross-reactivity, these goat anti-mouse IgG (H+L) whole secondary antibodies have been affinity purified and cross-adsorbed against bovine IgG, goat IgG, rabbit IgG, rat IgG, human IgG, and human serum. Cross-adsorption or pre-adsorption is a purification step to increase specificity of the antibody resulting in higher sensitivity and less background staining. The secondary antibody solution is passed through a column matrix containing immobilized serum proteins from potentially cross-reactive species. Only the nonspecific-binding secondary antibodies are captured in the column, and the highly specific secondaries flow through. The benefits of this extra step are apparent in multiplexing/multicolor-staining experiments (e.g., flow cytometry) where there is potential cross-reactivity with other primary antibodies or in tissue/cell fluorescent staining experiments where there are may be the presence of endogenous immunoglobulins. Anti-Mouse/Anti-Rabbit secondary antibodies are affinity-purified antibodies with well-characterized specificity for mouse/rabbit immunoglobulins respectively and are useful in the detection, sorting or purification of its specified target.

Animals and other research organisms

Policy information about studies involving animals; ARRIVE guidelines recommended for reporting animal research, and Sex and Gender in Research

Laboratory animals	*For laboratory animals, report species, strain and age OR state that the study did not involve laboratory animals.*
Wild animals	*Provide details on animals observed in or captured in the field; report species and age where possible. Describe how animals were caught and transported and what happened to captive animals after the study (if killed, explain why and describe method; if released, say where and when) OR state that the study did not involve wild animals.*
Reporting on sex	*Indicate if findings apply to only one sex; describe whether sex was considered in study design, methods used for assigning sex. Provide data disaggregated for sex where this information has been collected in the source data as appropriate; provide overall numbers in this Reporting Summary. Please state if this information has not been collected. Report sex-based analyses where performed, justify reasons for lack of sex-based analysis.*
Field-collected samples	*For laboratory work with field-collected samples, describe all relevant parameters such as housing, maintenance, temperature, photoperiod and end-of-experiment protocol OR state that the study did not involve samples collected from the field.*
Ethics oversight	*Identify the organization(s) that approved or provided guidance on the study protocol, OR state that no ethical approval or guidance was required and explain why not.*

Note that full information on the approval of the study protocol must also be provided in the manuscript.

Plants

Seed stocks	*Report on the source of all seed stocks or other plant material used. If applicable, state the seed stock centre and catalogue number. If plant specimens were collected from the field, describe the collection location, date and sampling procedures.*
Novel plant genotypes	*Describe the methods by which all novel plant genotypes were produced. This includes those generated by transgenic approaches, gene editing, chemical/radiation-based mutagenesis and hybridization. For transgenic lines, describe the transformation method, the number of independent lines analyzed and the generation upon which experiments were performed. For gene-edited lines, describe the editor used, the endogenous sequence targeted for editing, the targeting guide RNA sequence (if applicable) and how the editor was applied.*
Authentication	*Describe any authentication procedures for each seed stock used or novel genotype generated. Describe any experiments used to assess the effect of a mutation and, where applicable, how potential secondary effects (e.g. second site T-DNA insertions, mosiacism, off-target gene editing) were examined.*
